# LAMC2 promotes EGFR cell membrane localization and acts as a novel biomarker for tyrosine kinase inhibitors (TKIs) sensitivity in lung cancer

**DOI:** 10.1038/s41417-023-00654-7

**Published:** 2023-08-04

**Authors:** Dongdong Tong, Xiaofei Wang, Liying Liu, Ting Wen, QiaoYi Chen, Chen Huang

**Affiliations:** 1 Department of Cell Biology and Genetics, School of Basic Medical Sciences, Xi’an Jiaotong University Health Science Center, Xi’an, 710061 Shaanxi China; 2 Biomedical Experimental Center of Xi’an Jiaotong University, Xi’an, 710061 Shaanxi China; 3 Key Laboratory of Environment and Genes Related to Diseases, Xi’an Jiaotong University, Ministry of Education, Xi’an, 710061 China

**Keywords:** Targeted therapies, Cell biology

## Abstract

The epidermal growth factor receptor (EGFR) is one of the first and most prominent driver genes known to promote malignant lung cancer. Investigating regulatory mechanisms beyond ligand-receptor binding, phosphorylation, and receptor kinase activation as means of EGFR signaling activation is important for improving EGFR-targeted therapy. Here, we report that Laminin-5γ-2 (LAMC2) retained high oncogenic capacity in lung cancer, silencing LAMC2 inhibited EGFR-induced cell proliferation and tumor growth in vivo. Deletion mutation experiments showed that both the EGF-Lam and LamB regions of LAMC2 are necessary for EGFR receptor binding, and that LAMC2 and EGFR were found to co-localize at the endoplasmic reticulum (ER) membrane. In addition, LAMC2 overexpression enhanced EGFR membrane deposition and promoted EGFR transport from the ER. Moreover, LAMC2 was necessary for preventing EGFR protein degradation via ubiquitination. Lastly, our study showed that high LAMC2 expression is positively associated with response to gefitinib (EGFR tyrosine kinase inhibitor) treatment. Overall, our study revealed a new regulatory mechanism of LAMC2 in promoting EGFR protein expression and stability by facilitating ER transport and preventing protein degradation via ubiquitination. Moreover, LAMC2 may serve as a stratifying biomarker for patients suitable for EGFR-TKI treatment.

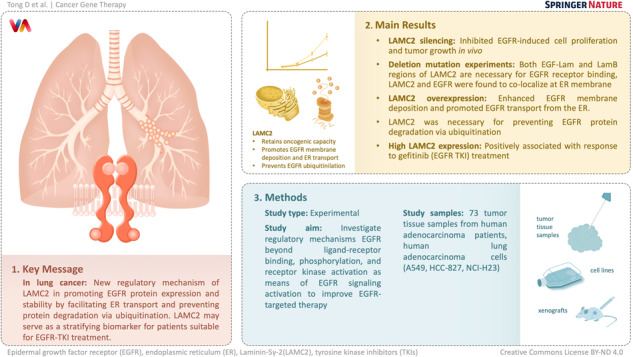

## Introduction

Epidermal growth factor receptor (EGFR) belongs to the HER/erbB family of receptor tyrosine kinases (RTKs), and is a prominent therapeutic target for non-small cell lung carcinomas (NSCLCs). Improper EGFR gain-of-function or activating mutation promotes constitutive TK activity, leading to stimulated cell proliferation, metastasis, angiogenesis, and reduced apoptosis [1, 2]. In fact, activating mutations in EGFR occur in 10–20% and 50% of Caucasian and Asian NSCLC patients, respectively. More than 85% of EGFR mutations are due to deletions in exon 19 and L858R missense mutation on exon 21. For lung cancer patients who are tested positive for activating EGFR mutations, tyrosine kinase inhibitors (TKIs) are now used as first-line treatment.

TKIs and monoclonal antibodies are agents specifically designed to reduce EGFR activity. To date, there are three generations of clinically approved TKIs [3]. The first generation (Erlotinib, Gefinitib, and Icotinib) TKIs inhibit EGFR activity by reversible binding to the ATP-binding site of EGFR kinase domain. The second generation (Afatinib and Dacominitib) TKIs primarily target tumors that have developed secondary resistance mutations by blocking ErbB3 transphosphorylation through irreversible and covalent bonding with ErbB family receptor homodimers and heterodimers. Lastly, the third and newest generation (Osimertinib, Rociletinib, Olmutinib, Lazertinib) TKIs specifically target T790M EGFR-mutant tumors, which occur in ~50% of patients who have developed resistance to first and second generation TKIs. Despite phenomenal drug response rates and overall improvement in survival, resistance to TKIs and continued disease progression usually occur 10–14 months post treatment [4]. In fact, patients having gone through all three generations of TKI treatment can still develop resistance. Thus, new research on mechanisms involved in elevated EGFR protein expression and signaling activation is necessary for improving therapeutic efficacy as well as finding new molecular targets.

Through systematic bioinformatic analyses to screen for significantly dysregulated genes based on drug sensitivity, our study found Laminin-5γ-2 (LAMC2) to be both upregulated and positively correlated with EGFR expression. Laminin-332 is a heterotrimeric glycoprotein and elemental constituent of the epithelial basement membranes, implicated in cell differentiation, migration, adhesion, and invasion. Three non-identical chains of laminin (α3, β3 and γ2) assemble in the endoplasmic reticulum (ER) via disulfide bonds to form Laminin-332 [5–7]. Missense mutation in α3(LAMA3) can lead to Laminin-322 accumulation within the endoplasmic reticulum (ER) [8, 9]. Aberrant LAMC2 expression is highly correlated with metastasis and poor prognosis in NSCLC patients [10–13]. However, the relationship between LAMC2 and EGFR in lung cancer remains elusive. Currently available research has reported that LAMC2 and EGFR expressions are positively correlated in bladder cancer, oral (squamous) carcinoma, cholangiocarcionma, as well as in several human cancer cell lines including breast, neoplastic, and ATC cell lines [11, 14–16]. In breast carcinoma, LAMC2 has been shown to bind EGFR and stimulate MAPK activation [17]. In addition, a study led by Xu et al. reported that in gastic cancer cells, LAMC2 can promote EGFR activation via phosphorylation [18]. EGFR signaling is highly dependent on the quantity of EGFR found at the cell membrane, which can be regulated via various mechanisms including EGFR protein synthesis, degradation, and transport. To date, no study has reported on whether LAMC2 can facilitate the protein transport and stability of EGFR to enhance its protein expression and signaling activation.

Our study aims to examine the role of LAMC2 in mediating EGFR protein expression and stabilization by facilitating its transport from the ER. We report that LAMC2 is an important EGFR TKI sensitive gene with oncogenic functions in lung cancer cells and mice xenograft. LAMC2 can directly bind to EGFR and promote its deposition at the ER membrane. In addition, we showed that BFA- and CHX-induced EGFR protein degradation via ubiquitination can be prevented by overexpressing LAMC2. Moreover, drug sensitivity assays showed that the effect of gefitinib in lung cancer cells and tumor tissues is dependent on LAMC2 expression. As an upstream regulator of EGFR, our data suggest that LAMC2 may serve as a novel biomarker for predicting positive response to EGFR TKIs treatment in NSCLC patients.

## Materials and methods

### Patient samples

All samples were obtained from The First Affiliated Hospital of Xi’an Jiaotong university, Xi’an, China. A total of 73 tumor tissue samples from human adenocarcinoma patients were used for the immunohistochemistry staining experiment. All patients were pathologically diagnosed with adenocarcinoma. Informed consent was obtained from all patients, and the study experiments/methods were approved by the Institute Research Ethics Committee of The Cancer Center of Xi’an Jiaotong University (No. 20221149).

### Animal experiments

Three-week-old male BALB/c-nude mice were purchased from Yaokang Biotech (Chengdu, China). The animals were housed 5 per cage at the Central Laboratory of Animal, Xi’an Jiaotong University Health Science Center under standard conditions (23 ± 2 °C, 40–70% humidity, ad libitum access to food and water, 12/12 h (light/dark) cycle, pathogen-free). The mice were observed for 1 week prior to the start of the experiments. For the first experiment, 15 mice were randomly divided into three groups (NC, LAMC2 KD, and LAMC2 KD + EGFR). 1 × 10^6^ A549 cells suspended in 0.1 ml PBS were subcutaneously injected into the right femoral area of each mouse. Tumor growth and volume were measured everyday from day 7 to day 35 using 3D IVIS Spectrum In Vivo Imaging System (PerkinElmer, MA, USA). At day 35, the mice were sacrificed via CO_2_, and the tumors were extracted through standard surgery. For the second experiment, 20 mice were randomly divided into four groups (Control, LAMC2, Control + Gefitnib, and LAMC2 + Gefitinib). 1 × 10^6^ A549 cells suspended in 0.1 ml PBS were subcutaneously injected into the right femoral area of each mouse. Tumor growth and volume were measured everyday from day 7 to day 35 using 3D IVIS Spectrum In Vivo Imaging System (PerkinElmer, MA, USA). Gefitnib (75 mg/kg) (MedChemExpress, Shanghai, China) suspended in saline water containing 0.05% Tween 80 was orally administered every day starting from day 15 to day 35. At day 34, the mice were sacrificed via CO_2_, and the tumors were extracted through standard surgery. For both experiments, tumor size was measured using Vernier calipers, and tumor volume was calculated according to the volume = length × (width^2^)/2 formula. GraphPad Prism 8.0 (GraphPad Software) was used to generate all graphs illustrating xenograft volume and weight. All animal experiments were approved by the Institutional Animal Care and Use Committee of Xi’an Jiaotong University, and all animals were handled according to institutional guidelines.

### Cell culture

Human lung adenocarcinoma A549 cells were obtained from Genechem (Shanghai, Genechem Co. Shanghai, China). Cells were cultured in F12K (Hyclone) with 10% fetal bovine serum (FBS, Biological Industries, Israel). Human lung adenocarcinoma HCC-827 cells were obtained from Genechem (Shanghai, Genechem Co. Shanghai, China). Cells were cultured in RPMI 1640 medium (Hyclone) with 10% fetal bovine serum (Biological Industries, Israel). Human lung adenocarcinoma NCI-H23 cells were obtained from Botian (Xi’an, China). All cells were cultured in RPMI 1640 medium (Hyclone) with 10% fetal bovine serum (Biological Industries, Israel). All cells were maintained in a 37 °C containing 5% CO_2_. Specific treatments of cells are listed below:

BFA treatment: cells were treated with BFA (MedChemExpress, Shanghai, China) dissolved in DMSO for 12 h at various doses (0, 5, 10, 20, and 25 μM).

MG132 treatment: cells were treated with MG132 (1 μM, MedChemExpress, Shanghai, China) dissolved in DMSO for 12 h.

Gefitinib treatment: cells were treated with unadulterated form of gefitinib (MedChemExpress, Shanghai, China) dissolved in DMSO for 12 h at various doses (0.1, 1, 3, 5, 10, 30, 60, 100, 200 μM).

CHX treatment: cells were treated with CHX (10 μg/ml, MedChemExpress, Shanghai, China) dissolved in DMSO for 6 h.

### Plasmid construction, siRNA synthesis, and cell transfection

siRNA was designed and synthesized by GenePharma (Shanghai, China). Non-sense siRNA was used as control. Transfection was carried out using Lipfectamine-2000 (Invitrogen, USA) according to manufacturer’s protocol. Sequences of shRNA and siRNA are listed in Supplementary Table 1. Knockdown and overexpression efficiency were validated using western blotting.

LAMC2 (WT), EGFR(WT) plasmids were purchased from Sino Biologica (Beijing, China). LAMC2 and EGFR domain deletion genes were amplified twice using overlap PCR Sequence of plasmids.

### Western blotting

Total protein was extracted using RIPA lysis buffer containing phosphatase and proteinase inhibitor (Beyotime, Beijing, China). Membrane protein was extracted using Membrane Protein Extraction Kit (Sangon Biotech, Shanghai, China). Protein concentrations were determined using bicinchoninic acid protein assay kit (Beyotime, Beijing, China). Equal concentrations of whole cell lysates were separated by 12% SDS-PAGE and transferred onto a nitrocellulose membrane, followed by blocking in 5% skim milk in TBST for 1 h at room temperature. The membranes were incubated with primary antibodies overnight at 4 °C, followed by HRP labeled secondary antibody for 1 h at room temperature. Protein bands were detected and analyzed using chemiluminescence and densitometric system. A complete list of antibodies used are listed in Supplementary Table 2.

### Cell cycle analysis

Cells were trypsinized, washed twice with ice-cold PBS, and fixed with 70% ethanol at 4 °C overnight. The cells were then washed and suspended in 150 μl RNase A (100 μg/ml) and 150 μl propidium iodide (50 μg/ml), and placed on ice for 15 min. Cell cycle analyses were performed using flow cytometry (FACSCalibur, BD, Biosciences, USA).

### Cell activity

Cells (5 × 10^3^) were plated into 96-well plates and cultured for 48 h. Cell activity was determined using CellTiter-Lumi™ luminescent cell viability assay kit (Beyotime, Beijing, China) at 570 nm absorbance using a microplate reader (POLARstar OPTIMA, BMG, Germany).

### Cell apoptosis

Cells were trypsinized and resuspended in 1 × binding buffer, and triple stained using the Hoechst (nuclei staining) and Annexin-V-FITC/PI Apoptosis Detection kit (Yeasen Biotechnology, Shanghai, China). Cells were double stained with Annexin V-FITC and PI solution successively for 15 min in the dark, and fixed with 4% paraformaldehyde for 20 min and Hoechst solution for 10 min in the dark. The cells were washed with PBS, and cell apoptosis was analyzed using flow cytometry (FACSCalibur, BD, Biosciences, USA).

### Colony formation

Cells were trypsinized and seeded in 6-well plates (500 cells/well). Cells were cultured for 10–14 days, and stained with 0.1% crystal violet solution (Sigma-Aldrich, USA) for 15 min. Colony numbers were analyzed using Quantity One Software (Bio-Rad, USA).

### Immunohistochemical staining

Formalin-fixed and paraffin-embedded tissue samples were cut into 5 μm thickness. The samples were deparaffinized using 100% xylene and hydrated using graded ethanol (100, 95, 80, and 70%). Antigen retrieval was performed in 0.01 M citrate buffer (pH 6.0), and the specimens were incubated with primary antibodies (EGFR, LAMC2, Phospho-EGFR, and Ki67) at 4 °C overnight. Immunodetection was performed using 3,3’-diaminobenzidine (DAB, Dako) and hematoxylin staining according to manufacturer’s instructions the following day. Images were obtained with Leica Microsystems (Leica, Germany). Intensity was manually scored. High expression was determined when more than 50% positive staining cells were detected in five randomly selected fields.

### Immunofluorescence microscopy

Cells were fixed with cold methanol and acetone for 20 min, washed three times with PBS, followed by blocking using 10% normal goat serum in PBS (0.3% Triton X-100) at room temperature for 1 h. Cells were then incubated with primary antibodies (LAMC2, EGFR, GRP78, His, or Flag) overnight at 4 °C, and fluorochrome-conjugated secondary antibody for 1 h at room temperature in the dark. After washing with PBS, 4’6-diamidino-2-phenylindole (DAPI) (0.1 mg/ml) (Beyotime, Beijing, China) was added. Immunofluorescent signals were detected using a fluorescence microscope (Leica, TCS SP8 DIVE, Germany).

### Growth inhibition assay

Cells were plated in 96-well plates (3–4000/well) in complete medium, and incubated for 24 h. The cells were then treated with Gefitnib (0.1–200 μM) dissolved in dimethyl sulfoxide (DMSO) for 24 h. cell viability was analyzed using CellTiter-Lumi™ luminescent cell viability assay kit (Beyotime, Beijing, China) at 570 nm absorbance using a microplate reader (POLARstar OPTIMA, BMG, Germany). IC_50_ was calculated as the concentration needed to achieve 50% reduction based on cell growth curves. Dose response curve fitting was performed using GraphPad Prism 8 (GraphPad).

### Co-IP assays

Total protein from cells were extracted using lysis buffer (150 mM NaCl, 1% NP-40, 50 mM Tris-HCl, pH8.0, protease inhibitor). Lysates were centrifuged at 13,000 × *g* at 4 °C for 10 min. Total cell lysates were then incubated with primary antibodies (anti-LAMC2, anti-LAMC2, anti-His, or anti-Flag) on Dynal magnetic beads (Invitrogen, CA, USA) overnight at 4 °C. The beads were then washed five times with PBS, and the immunoprecipitates were used for western blotting analysis.

### Protein docking

Full length LAMC2 (UniProt Q13753) and EGFR (UniProt P00533) PDB format files were downloaded from AlphaFold Protein Structure Database. Protein docking analysis was performed on https://cluspro.org/tut_dock.php.

### Statistics

All experiments were repeated three independent times, and data are presented as mean ± standard deviation. Student’s *t* test was used to determine the difference between two independent groups. *p* values < 0.05 were considered statistically significant. All data analyzation was performed using GraphPad Prism 8.0 (GraphPad Software).

## Results

### Systematic screening analyses identify LAMC2 as an EGFR TKI sensitive gene

**Fig. 1:**
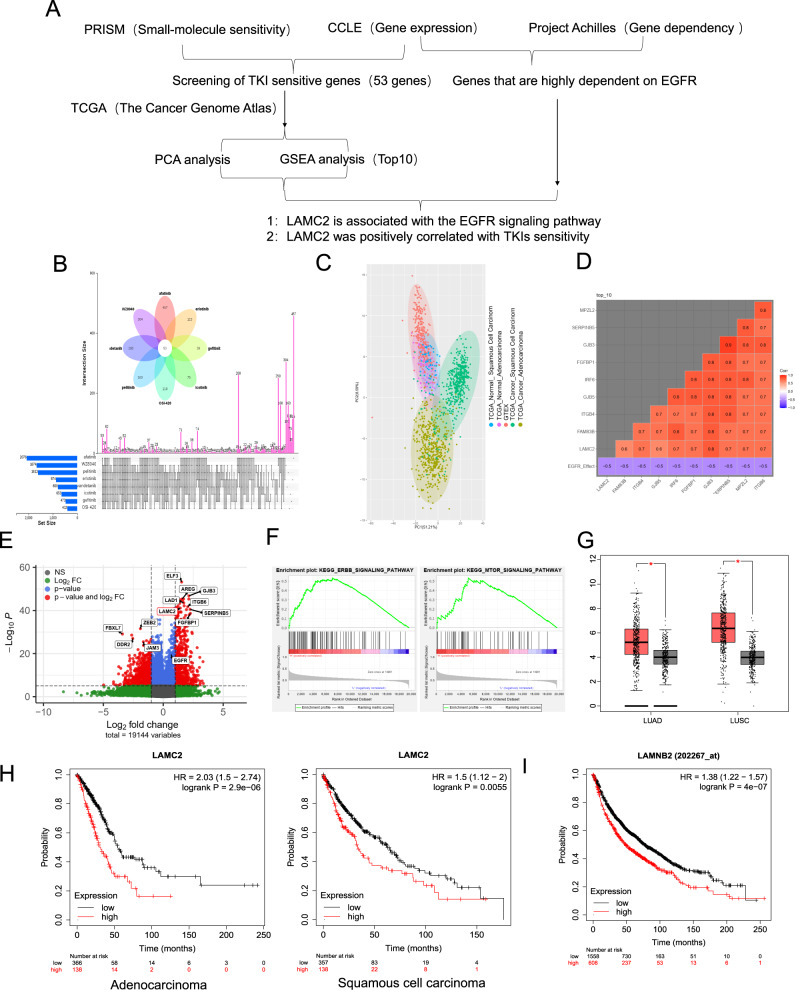
Systematic screening analyses identify LAMC2 as a key gene for promoting EGFR TKI sensitivity. **A** Bioinformatics analysis flowchart. **B** Cross-screening analyses using CCLE and PRISM identified 53 common genes significantly correlated with high TKI sensitivity in 930 different cancer cell lines. **C** Principal component analysis using TCGA database showed distinct clusters of these 53 genes in squamous cell carcinoma, adenocarcinoma, and their corresponding control groups. **D** Analysis using CCLE and Project Achilles data from DepMap (Cancer Dependency Map) portal showed correlation between top ten EGFR-dependent genes. **E** Volcano plot of gene effect analysis using CCLE and Project Achilles showed top differentially expressed genes associated with EGFR dependence (EGFR-dependent vs. EGFR-independent). **F** The gene set enrichment analysis (GSEA) indicate LAMC2 is significantly correlated with ERBB and MTOR signaling pathways in lung cancer. **G** TCGA data comparison of LAMC2 expression in lung adenocarcinoma (LUAD) and lung squamous cell carcinoma (LUSC) patient tumor and normal tissue samples. Red indicates tumor, and gray indicates normal tissue samples. **H** Kaplan–Meier survival probability curves of LUAD and LUSC patients (TCGA database) with low or high LAMC2 expression levels. **I** Kaplan–Meier survival probability curves of lung cancer patients with low or high LAMNB2 (LAMC2) expression levels (GEO database).

To identify genes integral for high EGFR TKI sensitivity, we used CCLE (Cancer Cell Line Encyclopedia) and PRISM (profiling relative inhibition simultaneously in mixtures) to cross-screen for common TKI sensitive genes. Eight EGFR TKIs were selected for this analysis: Afatinib, Erlotinib, Gefitinib, Icotinib, OSI-420, Pelitinib, Vandetanib, and WZ8040 (Fig. 1A). As shown in Fig. 1B, in 930 different cancer cell lines, 53 genes were found positively correlated with high TKI sensitivity. In addition, PCA analysis using TCGA database showed distinct clusters of these 53 genes in adenocarcinoma, squamous cell carcinoma, and their respective control groups (Fig. 1C and Supplementary Fig. 1A). This suggests that the 53 common drug sensitive genes may also serve as important driver genes for lung cancer. In addition, using CCLE and Project Achilles data from DepMap (Cancer Dependency Map) portal, we screened for top genes correlated with EGFR dependence in the same 930 cancer cell lines. And according to the database, a lower gene effect score indicates a higher gene dependence of cell proliferation. As shown in Fig. 1D, among the 53 common drug sensitive genes, the expression of MPZL2, SERPINB5, GJB3, FGFBP1, IRF6, GJB5, ITGB4, FAM83B, and LAMC2 were found positively correlated with high EGFR dependence. Top 30 differentially expressed EGFR-dependent genes are shown in Supplementary Fig. 1B. To corroborate this finding, based on EGFR gene effect score, 930 different cancer cell lines were divided into two groups (top 25% are EGFR-independent, bottom 25% are EGFR-dependent). We then used gene effect analysis to screen for genes associated with EGFR dependence. As shown in Fig. 1E, four genes (GJB3, SERPINB5, FGFBP1, LAMC2) from the top 10 correlated candidates in Fig. 1D were found in common in EGFR-dependent cancer cell types. To further examine whether any of the top 10 genes are correlated with ERBB and MTOR signaling pathways in lung cancer, we performed GSEA analysis using TCGA database. As indicated in Fig. 1F, LAMC2 was found correlated with ERBB as well as MTOR signaling. KEGG pathway enrichment analysis also showed that LAMC2 is significantly associated with small cell lung cancer and ERBB signaling pathway (Supplementary Fig. 1C). In addition, FGFBP1 was also found positively correlated with ERBB signaling (Supplementary Fig. 1D). TCGA and GEO database analyses showed that LAMC2 is highly expressed in both adenocarcinoma and squamous cell carcinoma patients, and its expression is negatively correlated with rate of survival (Fig. 1G–I). Overall, through multiple screening analyses using various databases, we were able to pinpoint LAMC2 as a key gene necessary for both EGFR TKI sensitivity as well as the development of lung cancer.

### LAMC2 promotes lung cancer cell proliferation and cell cycle disruption

**Fig. 2:**
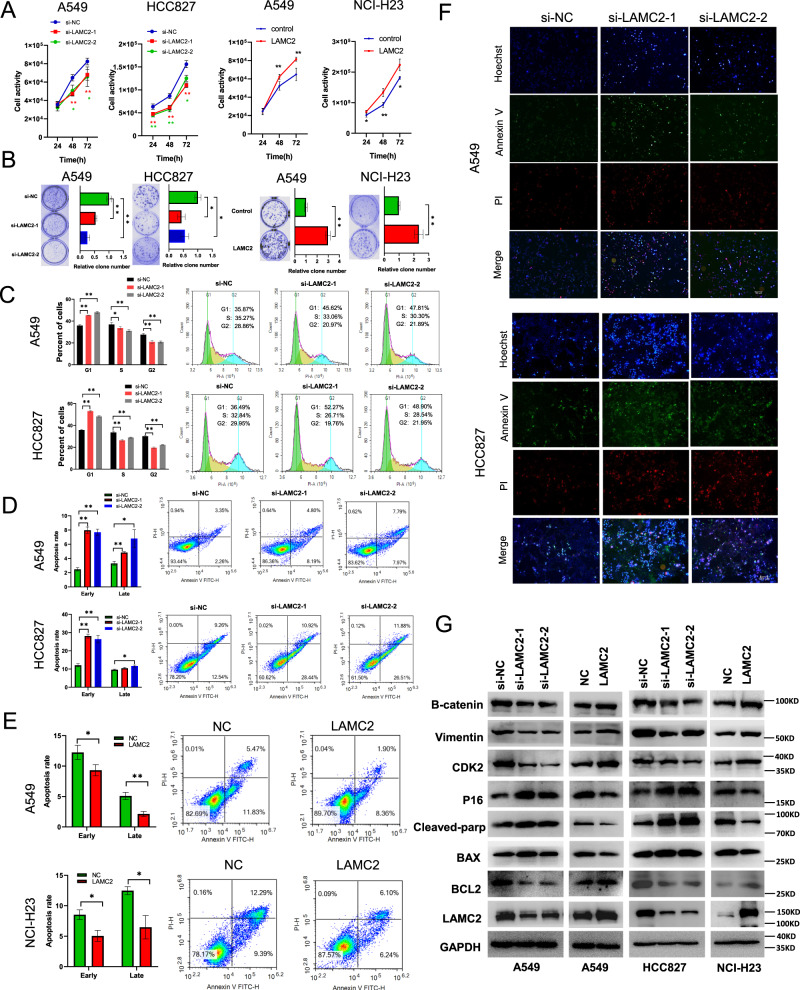
LAMC2 silencing inhibits lung cancer cell proliferation and cell cycle disruption. **A** Cell activity levels of A549, HCC827, and NCI-H23 cells transfected with either LAMC2 siRNA or LAMC2 overexpression vector at 24, 48, and 72 h. *p < 0.05, **p < 0.01. **B** Representative images of colony formation of A549, HCC-827, and NCI-H23 cell lines after LAMC2 siRNA or plasmid transfection. Quantification, right. *p < 0.05, **p < 0.01. **C** Flow cytometry of cell cycle progression in A549 and HCC827 cells after LAMC2 siRNA transfection, visualized by PI staining. *p < 0.05, **p < 0.01. **D**, **E** Flow cytometry of cell apoptosis in A549, HCC827 and NCI-H23 after transfection with LAMC2 siRNA or LAMC2 vector, visualized using Annexin-V/FITC-H. *p < 0.05, **p < 0.01. **F** Representative images of Hoechst, Annexin V, PI triple fluorescence staining showing A549 and HCC827 cell apoptosis after LAMC2 siRNA transfection. **G** Western blotting analyses indicating protein levels of B-catenin, vimentin, CDK2, P16, Cleaved-parp, BAX, BCL2 and LAMC2 in A549, HCC827, and NCI-H23 cells transfected with either LAMC2 siRNA and LAMC2 overexpression vector.

To establish the oncogenic capacity of LAMC2 in lung cancer, we generated both LAMC2 knockdown and overexpressing cell lines. Cell activity assays showed that LAMC2 knockdown in A549 and HCC827 cells significantly inhibited cell viability levels, while ectopic expression of LAMC2 promoted the activity levels of A549 and NCI-H23 cells (Fig. 2A). Moreover, LAMC2 knockdown reduced colony formation in A549 and HCC827 cells, while LAMC2 overexpression promoted colony growth in A549 and NCI-H23 cells (Fig. 2B). Next, we investigated the effect of LAMC2 on cell cycle control. As shown in Fig. 2C, flow cytometry results indicated that LAMC2 knockdown induced G1-phase cell cycle arrest, and concomitantly reduced the number of cells found in S and G2 phase. To corroborate this finding, we also showed that knockdown of LAMC2 in A549 and HCC827 cells significantly promoted both early and late apoptosis (Fig. 2D). On the contrary, rate of early and late apoptosis were substantially reduced by the overexpression of LAMC2 in A549 and NCI-H23 cells (Fig. 2E). In addition, we examined the effect of LAMC2 knockdown on cell apoptosis using Hoechst, Annexin V, and PI triple fluorescence staining. As shown in Fig. 2F, in contrast to si-NC cells, strong Annexin V fluorescence densities can be seen in si-LAMC2 cells. Western blotting analyses were also performed to examine potential changes in apoptosis-related proteins. As shown in Fig. 2G, LAMC2 is positively correlated with B-catenin, vimentin, CDK2, and BCL2 protein levels. Elevated protein levels of B-catenin, vimentin, and BCL2 have been evidenced to inhibit apoptosis and are frequently associated with epithelial mesenchymal transition (EMT) and tumor metastasis [19–21]. Moreover, inhibition of CDK2 in LAMC2 knockdown cells confirmed our cell cycle results which indicated G1 phase arrest. Western blotting analyses also revealed that LAMC2 knockdown elevated protein levels of P16, cleaved-parp, and BAX, which are associated with enhanced apoptosis [22–24]. Overall, results from this section highlight the tumor-promoting role of LAMC2 in lung cancer cells.

### LAMC2 silencing impedes EGFR-induced lung cancer cell proliferation

**Fig. 3:**
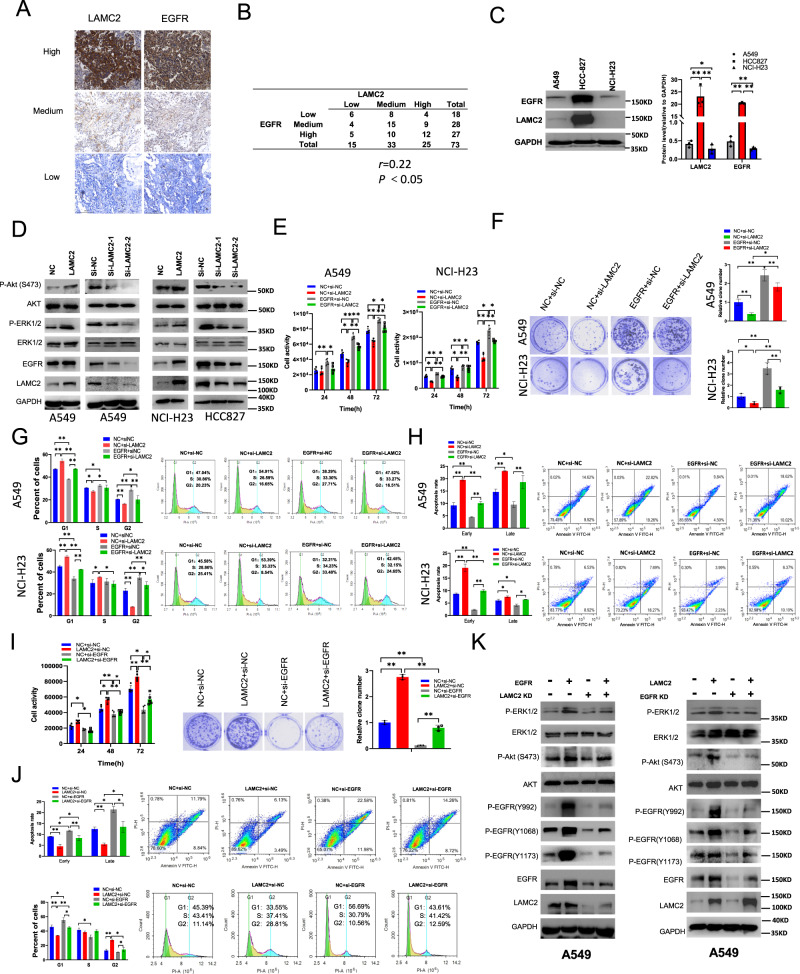
LAMC2 silencing inhibits EGFR-induced lung cancer cell proliferation and tumor growth. **A** Representative images of LAMC2 and EGFR immunostaining in human adenocarcinoma tissue samples. **B** Summary and correlation analysis of LAMC2 and EGFR immunostaining expression levels in adenocarcinoma tissue samples. *r* = 0.22, *p < 0.05. **C** Western blotting analyses indicating protein levels of EGFR and LAMC2 in A549, HCC827, and NCI-H23 cells. Quantification, right. *p < 0.05, **p < 0.01. **D** Western blotting analyses indicating protein levels of P-AKT, AKT, P-ERK1/2, ERK1/2, EGFR and LAMC2 in A549, NCI-H23 and HCC827 cells transfected with either LAMC2 siRNA or LAMC2 overexpression vector. **E** Cell activity levels of A549 and NCI-H23 cells co-transfected with NC + si-NC, NC + si-LAMC2, EGFR + si-NC, or EGFR + si-LAMC2 at 24, 48 and 72 h marks. *p < 0.05, **p < 0.01. **F** Representative images of colony formation of A549 and NCI-H23 cell lines after co-transfection with either LAMC2 siRNA, si-NC, or EGFR plasmid. Quantification, right. *p < 0.05, **p < 0.01. **G** Flow cytometry of cell cycle progression in A549 and NCI-H23 cells after co-transfection with NC + si-NC, NC + si-LAMC2, EGFR + si-NC, or EGFR + si-LAMC2. *p < 0.05, **p < 0.01. **H** Flow cytometry of cell apoptosis in A549 and NCI-H23 cells after co-transfection with NC + si-NC, NC + si-LAMC2, EGFR + si-NC, or EGFR + si-LAMC2. *p < 0.05, **p < 0.01. **I** Cell activity and colony formation assays in A549 cells co-transfected with NC + si-NC, NC + si-EGFR, LAMC2 + si-NC, or LAMC2 + si-EGFR. Quantification of colony formation assay, right. *p < 0.05, **p < 0.01. **J** Flow cytometry of cell apoptosis and cell cycle progression in A549 cells after co-transfection with NC + siNC, LAMC2 + si-NC, NC + si-EGFR, or LAMC2 + si-EGFR. *p < 0.05, **p < 0.01. **K** Western blotting analyses indicating protein levels of P-ERK1/2, ERK1/2, P-AKT(S473), AKT, P-EGFR(Y992), P-EGFR(Y1068) and P-EGFR(Y1173) in A549 cells after co-transfection with either EGFR overexpression vector and LAMC2 siRNA and/or A549 cells co-transfected with LAMC2 overexpression vector and/or EGFR siRNA.

Previous studies have reported the co-expression of LAMC2 and EGFR in various cancers including cholangiocarcinoma, anaplastic thyroid carcinoma, and bladder cancer [11, 14, 25]. To verify potential correlation between LAMC2 and EGFR2, we first examined protein levels of the two in adenocarcinoma 73 patient tissue samples using immunohistochemistry (IHC) (Fig. 3A, B). Specifically, of the 25 tumor samples showing high LAMC2 staining, 33 showed medium, and 15 showed low LAMC2 staining. Moreover, in the 27 tumor samples with high EGFR staining, 28 showed medium, and 18 showed low EGFR staining. These results suggest that protein expression of LAMC2 is significantly and positively correlated with that of EGFR. Similar results were also found in lung cancer cell lines. The protein level of EGFR parallels that of LAMC2, which is found highest in HCC827 cells, and lowest in NCI-H23 cells (Fig. 3C). These results confirm the hypothesis that LAMC2 and EGFR protein expressions are positively correlated in both lung cancer tissues and cell lines. We further examined the role of LAMC2 on the activation of EGFR-related signaling pathways such as those involving PI3K/AKT and RAS/MAPK. As shown in Fig. 3D, ectopic expression of LAMC2 in A549 and NCI-H23 cells enhanced EGFR protein expression, and promoted the activated (phosphorylated) forms of AKT and ERK1/2 proteins. Contrasting results were seen in LAMC2 knockdown A549 and HCC827 cells (Fig. 3D). These results suggest that LAMC2 knockdown inhibits EGFR protein expression as well as the activation of its downstream signaling pathways. In addition, we co-transfected A549 and NCI-H23 cells with EGFR overexpressing vector and LAMC2 siRNA to assess their potential counteracting effects on tumor cell activity, proliferation, cell cycle, and apoptosis. Our data revealed that LAMC2 knockdown inhibited while EGFR overexpression promoted cellular activities of A549 and NCI-H23 cells (Fig. 3E). More important, co-transfection of LAMC2 siRNA significantly reduced the effect of EGFR expression on cell activity levels. In addition, increased colony growth in A549 and NCI-H23 cells induced by EGFR overexpression was also shown to be inhibited by co-transfection of LAMC2 siRNA (Fig. 3F). Cell cycle analyses indicated that while EGFR overexpression inhibited G1 and promoted S/G2 phase arrest, co-transfection with si-LAMC2 significantly increased G1 and suppressed S/G2 arrest (Fig. 3G). Moreover, reduction in early and late apoptosis induced by EGFR overexpression was also restored by co-transfection with LAMC2 siRNA (Fig. 3H). To substantiate the above findings, we also co-transfected A549 cells with LAMC2 overexpressing vector and EGFR siRNA. As shown in Fig. 3I, enhanced cell activity and colony growth induced by LAMC2 overexpression was thwarted by co-transfection with EGFR siRNAs. Furthermore, while LAMC2 overexpressing A549 cells demonstrated reduced G1 and increased G2 cell cycle arrest as well as decreased early and late apoptosis, EGFR knockdown was able generate counteracting effects (Fig. 3J). In addition, we also examined the phosphorylation levels of ERK1/2, AKT, and EGFR when A549 cells were co-transfected with EGFR plasmid and LAMC2 siRNA or LAMC2 plasmid and EGFR siRNA. As shown in Fig. 3K, increased phosphorylation of ERK1, AKT, and EGFR induced by EGFR and LAMC2 overexpression was hindered by LAMC2 and EGFR knockdown, establishing a connection between LAMC2 and EGFR signaling pathways. Similar results were also shown in NCI-H23 cells (Supplementary Fig. 2C). Overall, these results suggested that not only are LAMC2 and EGFR positively correlated in protein expression levels, knocking down either of the two can directly hinder the tumor-promoting activity and function of the other.

### LAMC2 promotes lung tumor growth through the EGFR in vivo

**Fig. 4:**
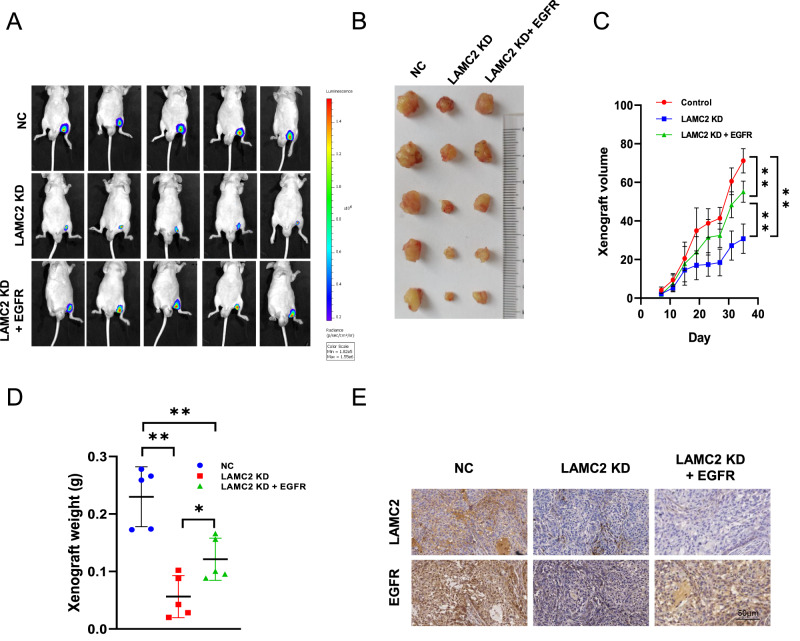
LAMC2 silencing inhibits EGFR-induced tumor growth in vivo. **A** Bioluminescence imaging of xenograft at day 35 in BALB/c-nude mice. Tumors were derived from subcutaneous injection of A549 (1 × 10^6^ cells suspended in 0.1 ml PBS) transfected with either NC, LAMC2 KD, or LAMC2 KD + EGFR. **B** Representative images of the gross morphology and size of tumors in NC, LAMC2 KD, and LAMC2 KD + EGFR groups. **C** Quantification of xenograft growth by volume every 3 days (from day 7 to days 35), right. *p < 0.05, **p < 0.01. **D** Quantification of xenograft weight after tumor extraction on day 35. *p < 0.05, **p < 0.01. **E** Representative images of IHC staining of xenograft tumor tissues for LAMC2 and EGFR.

To corroborate these results in vivo, we subcutaneously injected control, LAMC2-KD, as well as LAMC2-KD + EGFR A549 cells into 4-week-old male BALB/c-nude mice, and evaluated real-time tumor progression using fluorescence imaging (Fig. 4A). As shown in Fig. 4B–D, tumor size, volume, and weight were highest in the control group, and lowest in the LAMC2 knockdown group. Moreover, in mice co-transfected with both LAMC2 siRNA and EGFR plasmid, tumor size, volume, and weight were substantially reduced compared to the NC group. Lastly, IHC results of tumor tissues corroborated the relative protein expression levels of LAMC2 and EGFR in the three experimental groups in that LAMC2 and EGFR protein levels were highest in the NC group and lowest in the LAMC2 KD group (Fig. 4E).

### LAMC2 and EGFR bind to each other at the N-terminus

**Fig. 5:**
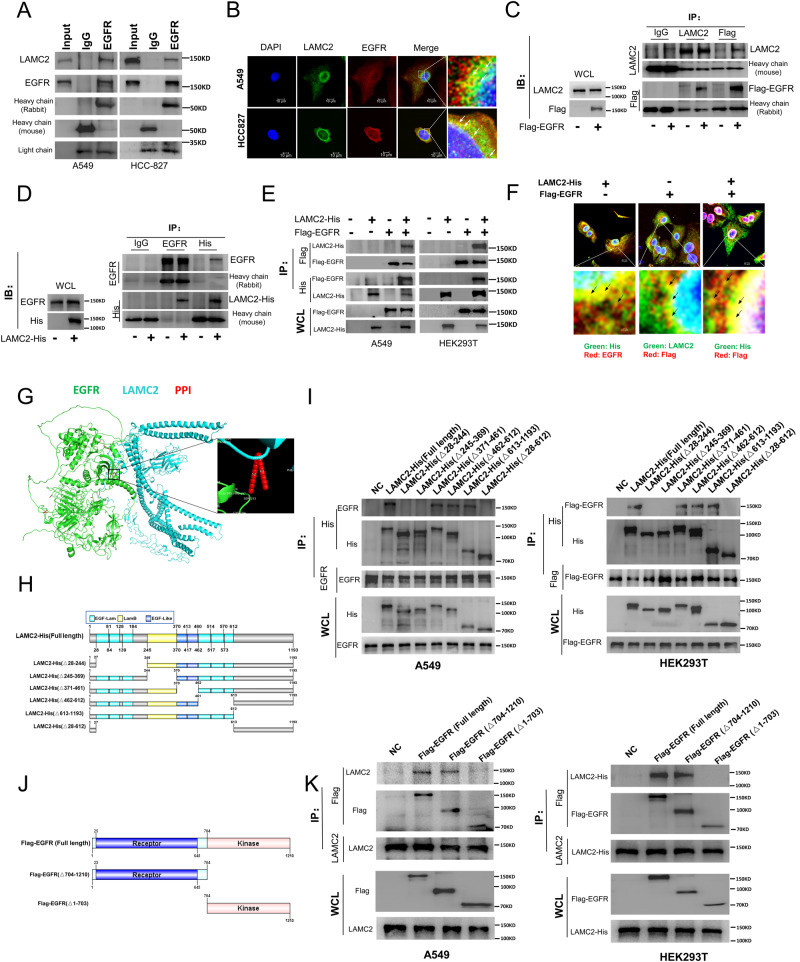
Binding and co-localization between LAMC2 and EGFR. **A** Co-immunoprecipitation (Co-IP) assays were used to detect association between EGFR and LAMC2 in A549 and HCC827 cells. **B** Representative images of immunofluorescence staining of DAPI, LAMC2, and EGFR in A549 and HCC827 cells showed co-localization between LAMC2 and EGFR near the nuclear membrane. **C** A549 cells were transfected with Flag-EGFR vector. Co-IP analyses were performed to detect protein interactions between exogenous EGFR and endogenous LAMC2. Immunoblot using WCL to detect LAMC2 and Flag, left. **D** A549 cells were transfected with LAMC2-His vector. Co-IP analyses were performed to detect protein interactions between exogenous LAMC2 and endogenous EGFR. Immunoblot using WCL to detect EGFR and His, left. **E** A549 and HEK-293T cells were co-transfected with LAMC2-His and Flag-EGFR expression vectors. Co-IP analyses were performed to detect protein interactions between exogenous LAMC2 and EGFR. **F** Representative images of immunofluorescence staining of His-tagged LAMC2 and Flag-tagged EGFR in A549 cells. Results indicated co-localization between His and EGFR, LAMC2 and Flag, as well as His and Flag near the nuclear membrane. **G** Representative image of protein–protein interaction complex. EGFR is shown in green, and LAMC2 is shown in blue. Protein–protein interaction positions marked by red. **H** Schematic representation of LAMC2 constructs with various domains removed. **I** A549 cells were transfected with different His-tagged LAMC2 vectors, while HEK-293T cells were co-transfected with Flag-EGFR and different His-tagged LAMC2 vectors. Western blotting analyses using WCL were performed to detect His, Flag and EGFR protein levels. Co-IP assays were used to detect associations between each vector and EGFR in A549, as well as interactions between different His-tagged LAMC2 and Flag-EGFR in HEK-293T cells. **J** Schematic representation of EGFR constructs with various domains removed. **K** A549 cells were transfected with different Flag-tagged EGFR, and HEK-293T cells were co-transfected with LAMC2-His and different Flag-tagged EGFR vectors. Western blotting analyses using WCL were performed to detect Flag, His and LAMC2 protein levels. Similarly, Co-IP assays were used to detect associations between each vector and LAMC2 in A549 cells, as well as interactions between different Flag-tagged EGFR and LAMC2-His in HEK-293T cells.

To further investigate the relationship between LAMC2 and EGFR, we proceeded to examine the location of LAMC2 and EGFR interaction in lung cancer cells. Co-immunoprecipitation (Co-IP) results confirmed direct protein binding between LAMC2 and EGFR in A5549 and HCC-827 cells (Fig. 5A). Moreover, immunofluorescence was used to examine the location of LAMC2 and EGFR binding in the cell. As shown in Fig. 5B, LAMC2 and EGFR co-localized near the nuclear membrane. As a type I transmembrane protein, EGFR has been evidenced to insert into the ER membrane, which is contiguous with the nuclear membrane [26]. Additional Co-IP analyses were performed using Flag-tagged EGFR and His-tagged LAMC2 to confirm their interaction. As shown in Fig. 5C, D, ectopically expressed Flag-EGFR directly interacted with LAMC2, and ectopically expressed LAMC2-His was also capable of interacting with EGFR. In addition, interaction between LAMC2-His and Flag-EGFR was also confirmed in A549 and HEK293T cells (Fig. 5E). Moreover, co-localization between LAMC2-His and Flag-EGFR near the nuclear membrane was validated using immunofluorescence assays (Fig. 5F). Previously, LAMC2 and EGFR were only found to co-localize on the cell membrane in anaplastic thyroid carcinoma cells [11]. Our results provide the first indication that LAMC2 and EGFR can co-localize at the nuclear membrane. To predict the binding region between LAMC2 and EGFR, we performed protein docking analysis using Cluspro. As shown in Fig. 5G, LAMC2 and EGFR is predicted to bind at the N-terminal region. While it has been previously reported that recombinant domain III of LAMC2 can bind to EGFR, the exact binding sites between the two remain unclear. To identify the specific LAMC2-binding region for EGFR, six deletion mutants (serial deletion of the EGF-Lam, LamB, and EGF-Like regions) of LAMC2 were constructed (Fig. 5H). Co-IP and immunoblotting results revealed that both EGF-Lam (28–244) and LamB (245–369) regions of LAMC2 were required to bind EGFR, while EGF-Like (271–461), EGF-Lam (462–612), and C-terminal (613–1193) were not (Fig. 5I). In addition, we also constructed two deletion mutants (receptor and kinase regions) for EGFR (Fig. 5J). Results from Co-IP and immunoblotting showed that the receptor region of EGFR is responsible for LAMC2 binding, while the kinase region is not (Fig. 5K). These results revealed that both the EGF-Lam and LamB regions of LAMC2 are required for EGFR receptor binding. EGFR protein can be expressed in the soluble form due to lack of membrane localization signal at the amino terminus [27]. Our data (Fig. 5J, K) confirmed that when the amino terminus is deleted from EGFR, LAMC2 was unable to bind. On the other hand, intact EGFR as well as truncated form with retention of the amino terminus were both able to bind to LAMC2. This result suggests that LAMC2 specifically targets membrane-associated EGFR and is important for promoting its membrane localization. Collectively, our data showed that LAMC2 not only interacts with EGFR at the cell membrane as previously reported, but also at the nuclear membrane, which reveals potential new regulatory mechanisms between the two.

### LAMC2 is essential for EGFR membrane deposition, protein stability, and ER transport

**Fig. 6:**
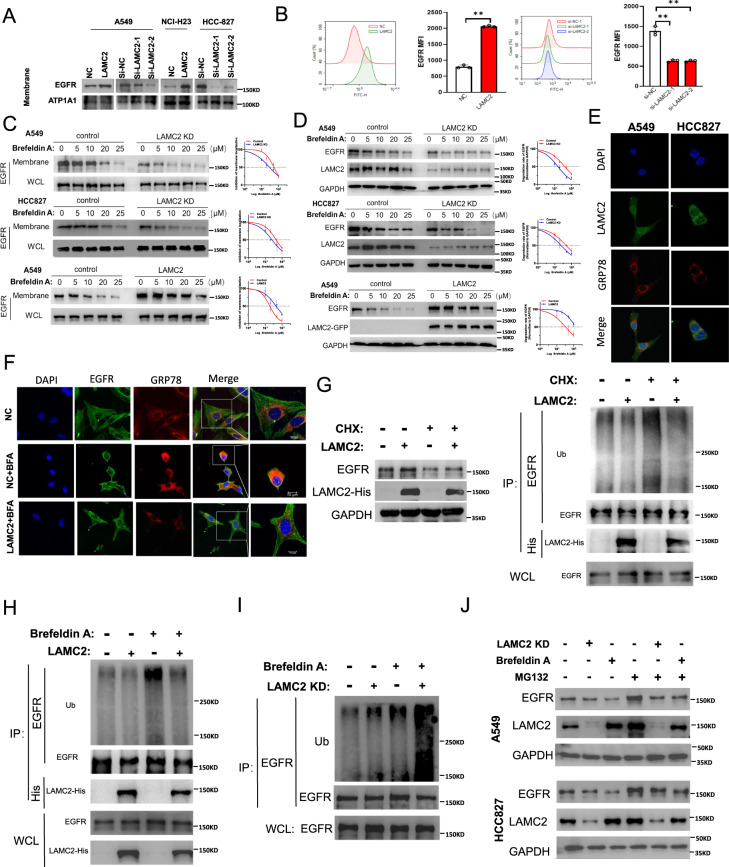
EGFR protein stabilization and deposition at the endoplasmic reticulum membrane is mediated by LAMC2. **A** Western blotting analyses indicating membrane EGFR protein expression levels in LAMC2 knockdown or overexpressing lung cancer cell lines. **B** EGFR membrane luminescence measured by flow cytometry. *p < 0.05, **p < 0.01. **C** EGFR protein levels found in the membrane and whole cell lysates (WCL) of control and LAMC2 knockdown A549 and HCC827 cells, as well as LAMC2 overexpressing A549 cells subjected to BFA treatment. Quantification of inhibition of EGFR membrane localization, right. **D** Western blotting analyses indicating protein levels of EGFR and LAMC2 in control and LAMC2 knockdown A549 and HCC827 cells, as well as LAMC2 overexpressing A549 cells subjected to BFA treatment. Quantification of EGFR half‐life changes, right. **E** Representative images of immunofluorescence staining of DAPI, LAMC2, and GRP78 showed co-localization between LAMC2 and GRP78 at the nuclear/ER membrane. **F** Representative images of immunofluorescence staining of DAPI, EGFR, and GRP78 in NC and LAMC2 overexpressing A549 cells subjected to BFA treatment. **G** A549 cells were transfected with LAMC2-His and subjected to CHX treatment (10 μg/ml). Protein extracts were subjected to co-immunoprecipitation, ubiquitination, and immunoblot assays. **H** A549 cells were transfected with LAMC2-His and subjected to BFA treatment. Protein extracts were subjected to co-immunoprecipitation, ubiquitination, and immunoblot assays. **I** A549 LAMC2 knockdown cells were subjected to BFA treatment. Protein extracts were subjected to co-immunoprecipitation, ubiquitination, and immunoblot assays. **J** A549 cells were subjected to LAMC2 knockdown, BFA, and/or MG132 (1 μM) treatments. Western blotting analyses were performed to assess protein levels of EGFR and LAMC2.

Next, we investigated the role of LAMC2 in regulating EGFR protein expression. First, we examined the effect of LAMC2 expression on EGFR membrane localization. Western blotting analyses showed that LAMC2 overexpression enhanced membrane EGFR protein levels in A549 and NCI-H23 cells (Fig. 6A). Conversely, LAMC2 knockdown in A549 and HCC-827 cells significantly inhibited membrane EGFR protein levels (Fig. 6A). Moreover, flow cytometry analyses demonstrated that LAMC2 expression is positively correlated with EGFR membrane luminescence, confirming the role of LAMC2 in promoting membrane EGFR protein levels in A549 cell line (Fig. 6B). Overall, these results suggest that LAMC2 is essential for regulating EGFR membrane protein levels. To explore the mechanism of EGFR protein regulation via LAMC2, we ascertained whether LAMC2 is involved in the transport of EGFR from the ER. LAMC2 knockdown and overexpressing A549 and HCC827 cells were treated with Brefeldin A (BFA), an ER to Golgi protein transport inhibitor. Previous studies have reported that BFA treatment can prevent normal protein secretion and induce ER stress due to accumulation of proteins within the ER [28, 29]. Our results indicated that treatment of BFA resulted in a dose-dependent decrease in EGFR membrane localization in both control, LAMC2 knockdown, and overexpressing cells (Fig. 6C). Interestingly, while LAMC2 knockdown cells showed greater reduction in EGFR membrane localization than the corresponding control cells, LAMC2 overexpressing cells showed the exact opposite. As our data indicated, BFA treatment and LAMC2 knockdown induced synergistic effect in diminishing EGFR membrane localization, and that overexpressing LAMC2 provided rescuing effects. This suggests that LAMC2 can counter the effects of BFA and promote EGFR transport from the ER. Western blotting analyses were performed to examine the protein expression levels of EGFR and LAMC2 under BFA treatment. As shown in Fig. 6D, BFA treatment led to a dose-dependent decrease in EGFR and increase in LAMC2 protein levels in both control and LAMC2 knockdown cells. Increase in LAMC2 protein expression post BFA treatment may be an adaptive response to EGFR accumulation; however, further validation is needed. In addition, compared to the control group, LAMC2 overexpressing cells provided rescuing effects on EGFR protein levels. Collectively, these data suggest that LAMC2 is capable of countering the effect of BFA to facilitate EGFR ER transport and promote membrane deposition.

Given evidence of LAMC2 in promoting EGFR transport from the ER, we subsequently performed immunofluorescence assays to confirm whether LAMC2 and EGFR can be found in the ER. In A549 and HCC-827 cells, LAMC2 and glucose-regulated protein 78 (GRP78), a molecular chaperone essential for ER stress response, were shown to co-localize at the ER membrane (Fig. 6E). In addition, we also showed that EGFR can co-localize with GRP78 at the ER membrane. Moreover, while BFA treatment diminished EGFR protein deposition and co-localization with GRP78 at the ER membrane, LAMC2 overexpression was able to provide rescuing effects (Fig. 6F). Overall, our data confirm that LAMC2 interacts with EGFR to promote its transport from the ER thereby enhancing its membrane deposition.

To examine whether LAMC2 enhances EGFR protein expression by preventing protein degradation, we assessed EGFR protein expression levels when treated with cycloheximide (CHX), a protein synthesis inhibitor. As Fig. 6G indicated, while treatment with CHX led to a reduction in EGFR protein levels, overexpression of LAMC2 was able to reverse this effect. Additionally, Co-IP analyses showed that LAMC2 overexpression can directly hinder CHX-induced EGFR ubiquitination, which implies that LAMC2 can prevent EGFR protein degradation via ubiquitination (Fig. 6G). Moreover, our data also demonstrated that LAMC2 overexpression significantly suppressed BFA-induced EGFR ubiquitination, while LAMC2 knockdown along with BFA treatment induced a synergistic increase in EGFR ubiquitination levels (Fig. 6H, I). Lastly, we showed that treatment with MG132 significantly promoted EGFR protein levels in LAMC2 knockdown and BFA-treated cells (Fig. 6J). Noted, the effect of MG132 treatment in promoting EGFR protein levels was more apparent in HCC827 cells than in A549 cells. As a proteasome inhibitor, MG132 can reduce degradation of ubiquitin-conjugated proteins. Hence, this suggests that low LAMC2 may be responsible for EGFR accumulation within the ER and its subsequent protein degradation through ubiquitination. Taken together, our data provided a clear indication that LAMC2 is able to prevent EGFR ubiquitination induced by both CHX and BFA treatments. This also signifies that LAMC2 enhances EGFR protein expression by facilitating its transport from the ER and preventing ER-associated protein degradation via ubiquitination.

### Gefitinib sensitivity is dependent on LAMC2 expression

**Fig. 7:**
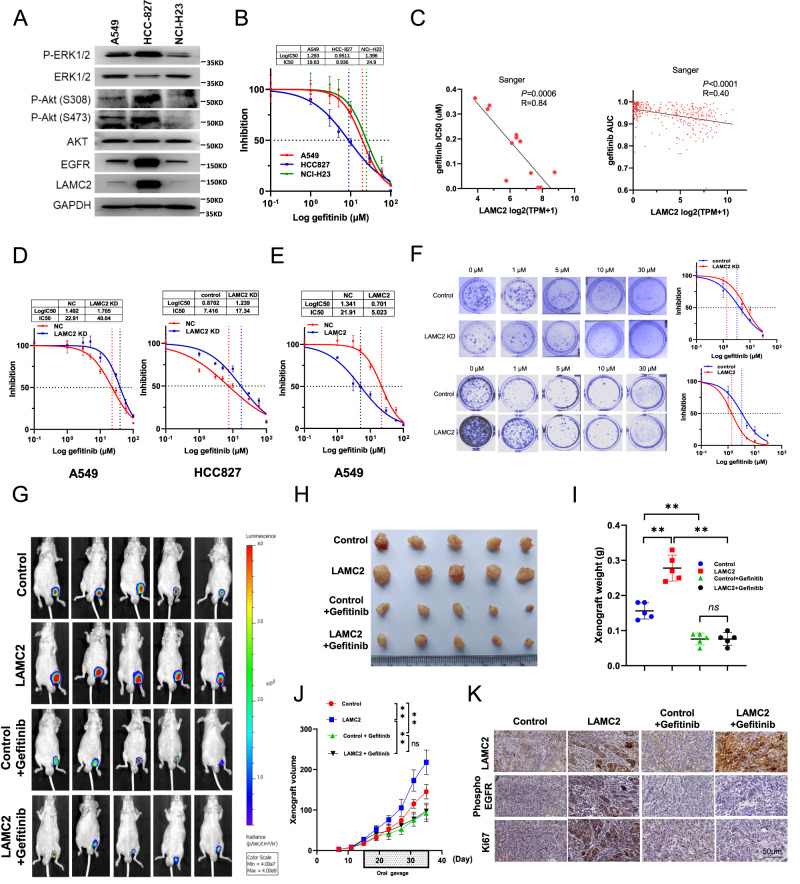
Gefitinib sensitivity is dependent on LAMC2 expression. **A** Western blotting analyses indicating protein levels of P-ERK1/2, ERK1/2, P-Akt(S308/S473), AKT, EGFR and LAMC2 in A549, HCC827 and NCI-H23 cells. **B** The 50% inhibitory concentration (IC50) values of gefitinib in A549, HCC-827 and NCI-H23 cells at various doses (0.1, 1, 3, 5, 10, 30, 60, 100, 200 μM). **C** Sanger database correlation analysis of LAMC2 expression with IC50 of gefitinib (Left, *R* = 0.84, *p* = 0.0006) and AUC (Right, *R* = 0.40, *p* < 0.0001). **D** IC50 values of gefitinib in stable LAMC2 knockdown A549 and HCC827 cells. **E** IC50 values of gefitinib in stable LAMC2 overexpression A549 cell. **F** Representative images of colony formation of control, stable LAMC2 knockdown or overexpression A549 cells subjected to gefitinib treatment. Quantification, left. *p < 0.05, **p < 0.01. **G** Bioluminescence imaging of xenograft at day 35 in BALB/c-nude mice. Tumors were derived from subcutaneous injection of A549 (1 × 10^6^ cells suspended in 0.1 ml PBS) transfected with either control or LAMC2 plasmid subjected to gefitinib treatment (75 mg/kg). **H** Representative images of the gross morphology and size of tumors in NC, LAMC2, control + Gefitinib, and LAMC2 + Gefitinib groups. **I** Quantification of xenograft weight after tumor extraction on day 35. *p < 0.05, **p < 0.01. **J** Quantification of xenograft growth by volume every 3 days (from day 7 to days 35), right. *p < 0.05, **p < 0.01. **K** Representative images of IHC staining of xenograft tumor tissues for LAMC2, Phospho-EGFR, and Ki67.

Previous sections of this study demonstrated that LAMC2 promoted EGFR protein stability through direct binding and facilitation of its transport from the ER membrane. Moreover, bioinformatics analyses showed that LAMC2 expression is predictive of higher sensitivity to EGFR-targeted TKIs. To verify the above findings, we examined the importance of LAMC2 expression on TKI sensitivity both in vitro and in vivo. First, western blotting was performed to examine potential correlation between LAMC2 expression and EGFR-related signaling pathways such as those involving ERK and AKT. As shown in Fig. 7A, high LAMC2 and EGFR protein expressions are positive correlated with increased phosphorylation of ERK1/2 and AKT. In addition, we tested cell sensitivity to gefitinib, a widely used lung cancer treatment, by examining the 50% inhibitory concentration (IC50), which is traditionally used to determine drug sensitivity and potency. Noted, gefitinib has been shown to increase the formation of inactive EGFR dimers through communication between the kinase domain and the extracellular dimerization domain, suggesting the possibility that gefitinib‐induced dimers could be more rapidly endocytosed and degraded [30, 31]. Results showed that IC50 is lowest in HCC827 cells, followed by A549, and NCI-H23 cells (Fig. 7B). This suggests that high LAMC2 and EGFR protein expressions are indicative of high gefitinib sensitivity. Bioinformatics analyses using the Sanger database were also performed. We found that gefitinib IC50 and AUC were inversely correlated with LAMC2 expression, which confirmed our findings in A549, HCC827, and NCI-H23 cell lines (Fig. 7C). Sensitivity of lung tumor cells to TKI drugs depends on the level of EGFR signaling pathway-dependence. As we previously demonstrated, LAMC2 facilitates EGFR localization at the cell membrane, thereby enhancing EGFR protein stability, which is essential for promoting lung cancer cell growth. This also explains why LAMC2 expression is positively correlated with high EGFR expression and gefitinib sensitivity. To tested the effect of LAMC2 knockdown and overexpression on gefitinib sensitivity using IC50 and cell activity assays, stable LAMC2 knockdown and overexpression were constructed in A549 and HCC827 cells. As our results indicated, drug sensitivity and cell activity were especially low in LAMC2 stable knockdown A549 and HCC827 cell lines, and high in LAMC2 stable overexpressing A549 cell lines (Fig. 7D, E and Supplementary Fig. 3A–C). In addition, we showed that while gefitinib was able to inhibit colony growth in both LAMC2 stable knockdown and overexpression cells, sensitivity to the drug was significantly lower in the LAMC2 stable knockdown group, and stable overexpression cell line shown higher sensitivity (Fig. 7F). Together, these results suggest that gefitinib may selectively target LAMC2 in lung cancer cells.

To validate results found in vitro, we also tested the effect of gefitinib in reducing tumor growth in nude mice treated with either control or LAMC2 overexpressing A549 cells (Fig. 7G). As shown in Fig. 7H–J, tumors in the LAMC2 overexpression group were significantly larger in size and higher in weight and volume compared to the control group. Interestingly, tumor size, weight, and volume were similar between the control + Gefitinib and LAMC2 + Gefitinib groups. This suggests that the tumor reduction effect of gefitinib was extremely effective in the LAMC2 overexpression group. Lastly, IHC analyses of tumor tissues indicated that Gefitinib effectively reduced Phospho-EGFR and Ki67 protein levels in both control and LAMC2 groups (Fig. 7K). However, under Gefitinib treatment, the degree of Phospho-EGFR and Ki67 protein reduction was more significant in the LAMC2 group compared to the control group. This suggests that sensitivity to Gefitinib is higher when LAMC2 expression is high. Overall, these results signify that gefitinib may selectively target LAMC2 both in vitro and in vivo, and suggest that LAMC2 may serve as an effective therapeutic target for EGFR TKI drugs.

## Discussion

In this study, through systematic screening analyses, we report that LAMC2 is an EGFR TKI sensitive gene with both oncogenic and protein transport functions in lung cancer. As a subunit of laminin-332, which is responsible for normal epithelial cell adhesion, differentiation, and migration, LAMC2 is frequently overexpressed in various cancers including, lung cancer [12, 13, 32, 33], laryngeal cancer [34], gastric cancer [18], cholangiocarcinoma [25], thyroid carcinoma [11], and pancreatic ductal adenocarcinoma [35]. In accordance with previous reports, our results indicated that LAMC2 silencing inhibited lung cancer cell progression through reduced cell activity, increased apoptosis, arrest in G1 to S cell cycle progression, and diminished tumor growth in vivo. Western blotting results also revealed the ability of LAMC2 to promote vimentin protein expression, which is highly correlated with the occurrence of metastasis and poor prognosis in lung cancer patients [36–39]. Overall, these results substantiate the oncogenic capacity of LAMC2 in lung cancer. Currently reported underlying mechanisms of LAMC2-induced cancer cell progression and metastasis include regulation of integrin B1-, ZEB1, and Snail expression [13], as well as activation of AKT1 and EGFR [11, 14, 18, 25, 32, 35]. Based on our findings, we showed that LAMC2 and EGFR co-expressed in lung cancer cells and tissues. Co-expression of LAMC2 and EGFR have also been reported in bladder cancer, anaplastic thyroid carcinoma, and cholangiocarcinoma [11, 14, 25]. For example, in anaplastic thyroid carcinoma, LAMC2 has been reported to bind EGFR to promote EGFR inactivation [11]. Co-IP experiments have also confirmed direct binding between LAMC2 and EGFR in PDAC cells [35]. Moreover, knockdown of LAMC2 was able to inhibit EGFR phosphorylation as well as its downstream targets ERK and AKT in pancreatic, anaplastic thyroid carcinoma, and gastric cancer cells [11, 18, 35]. Corroborating previous findings in other cancer cell lines, our results indicated that LAMC2 expression promoted the phosphorylation of EGFR, as well as its downstream targets AKT and ERK1/2 in A549, NCI-H23, and HCC827 lung cancer cells. However, it is important to note that a negative feedback loop between LAMC2 expression and AKT phosphorylation has been previously reported in PC-9 and H358 cells [32], while in our study LAMC2 expression promoted AKT phosphorylation at S473. The contradictory results may be due to differences in cell lines used. Moreover, we showed that silencing of either LAMC2 or EGFR generated direct opposite effects on colony growth, cell cycle progression, cell activity, apoptosis, and tumor growth. These results established the role LAMC2 to enhance EGFR protein expression and signaling activation. In addition, silencing of LAMC2 inhibited the oncogenic capacity of EGFR in lung cancer cells, suggesting that LAMC2 acts upstream of EGFR. While previous studies have focused on the role of LAMC2 in activating EGFR through phosphorylation, we have proposed a novel ability of LAMC2 to act as an EGFR protein transport facilitator. More important, through Co-IP and immunofluorescence analyses, our data showed that LAMC2 and EGFR co-localized at the ER membrane. Specifically, mutant deletion experiments have defined that the receptor region of EGFR and EGF-Lam (1–244)/LamB (245–369) regions of LAMC2 were responsible for their protein binding.

To understand how LAMC2 promotes EGFR protein expression and signaling, and why they co-localize at the ER membrane, we first evaluated the effect of LAMC2 expression on membrane EGFR protein expression. Through western blotting and flow cytometry analyses, we demonstrated that LAMC2 expression significantly increased EGFR membrane protein expression. More important, LAMC2 was able to counter BFA-induced reduction in EGFR membrane and total protein expression. As a prominent inhibitor of ER to Golgi protein transport, BFA can lead to ER and Golgi stress due to rapid cytotoxic accumulation of proteins in the ER, which would otherwise be transported to the Golgi for processed secretion [28, 29]. The ability of LAMC2 expression to prevent BFA-induced decline in EGFR protein expression suggests that LAMC2 may function to facilitate and enable EGFR protein transport from the ER. Not surprisingly, immunofluorescence analyses demonstrated the co-localization between GRP78 and LAMC2, as well as EGFR at the ER membrane. In addition, we also showed that BFA treatment inhibited, while LAMC2 overexpression enhanced EGFR membrane deposition and co-localization with GRP78. As a molecular chaperone in the ER, GRP78 is mainly responsible for protein folding and assembly, proteasome degradation of misfolded proteins, and ER stress sensor activation [40, 41]. Moreover, in the case of ER stress, GRP78 is able to activate the unfolded protein response (UPR) and restore cellular homeostasis via induced cell death. Interestingly, GRP78 overexpression has been demonstrated to be involved in lung cancer invasion and metastasis [41–43]. It is difficult to visually separate the nuclear membrane from the ER membrane since the tubular organization of ER allows it to extend through the entire cell [44]. However, the occurrence of LAMC2 and EGFR co-localization with GRP78 provides an indication that LAMC2 and EGFR protein binding may take place at the ER membrane. It has been demonstrated that abnormal EGFR expression in cancer is in part due to dysregulated protein transport [45]. To assess the hypothesis that LAMC2 promotes EGFR protein expression and stability through facilitating its protein transport from the ER, we examined the ability of LAMC2 to prevent EGFR protein degradation via ubiquitination. Under normal circumstances, to avoid cytotoxic accumulation within the ER, proteins that are misfolded or failed to be incorporated and transported out of the ER, are translocated into the cytosol for subsequent ubiquitination and degradation [46]. As our results have shown, treatment of BFA and CHX, a potent inhibitor of protein synthesis, both led to an increase in EGFR ubiquitination while LAMC2 overexpression generated rescuing effects. In addition, combination effect of BFA and LAMC2 knockdown led to synergistic enhancement of EGFR ubiquitination and reduction in protein expression. Moreover, treatment of MG132, a proteasome inhibitor, significantly improved BFA and LAMC2 knockdown-induced EGFR protein reduction. Taken together, our data revealed that LAMC2 binds to EGFR at the ER membrane to facilitate its transport and prevent its degradation via ubiquitination.

Having demonstrated the oncogenic capacity of LAMC2 in lung cancer as well as its ability to enhance EGFR expression and signaling through facilitating protein transport and stabilization, we went on to assess the importance of LAMC2 expression on EGFR TKI sensitivity. Previously, LAMC2 has been reported to serve as a potential therapeutic target of cetuximab in laryngeal cancer [34]. Cetuximab is an anti-EGFR monoclonal antibody, and has been found to effectively treat laryngeal cancer. To the best of our knowledge this is the first study to examine the effect of LAMC2 expression on EGFR TKI sensitivity. Our findings showed that the effectiveness of gefitinib to reduce cancer cell activity, colony growth, and tumor growth in vivo was highly dependent on LAMC2 expression levels. Specifically, high LAMC2 expressing cells showed higher sensitivity to gefitinib treatment compared to cells with silenced LAMC2 expression. Moreover, gefitinib significantly inhibited LAMC2 and EGFR protein expression levels. As an upstream regulator of EGFR, LAMC2 not only predicts high gefitinib sensitivity, but may also serve as a potential biomarker for predicting EGFR TKI response as well as for identifying and stratifying LAMC2^high^-EGFR^high^ NSCLC patients for EGFR TKI treatment. Overall, our study highlighted a novel regulatory function of LAMC2 in facilitating EGFR transport from the ER to enhance its protein expression and signaling. Future studies may also want to explore the effectiveness of targeting both LAMC2 and EGFR to enhance therapeutic efficacy.

### Supplementary information

wb raw data

Supplementary Tables

Supplementary Figures

## Data Availability

All data generated or analyzed during this study are available from the corresponding author on reasonable request.
