# Supplementary material for: LAMC2 promotes EGFR cell membrane localization and acts as a novel biomarker for tyrosine kinase inhibitors (TKIs) sensitivity in lung cancer
Source: Cancer Gene Ther. 2023 Aug 4;30(11):1498–512. doi: 10.1038/s41417-023-00654-7 (PMC10645587; doi:10.1038/s41417-023-00654-7)

Figure 2G


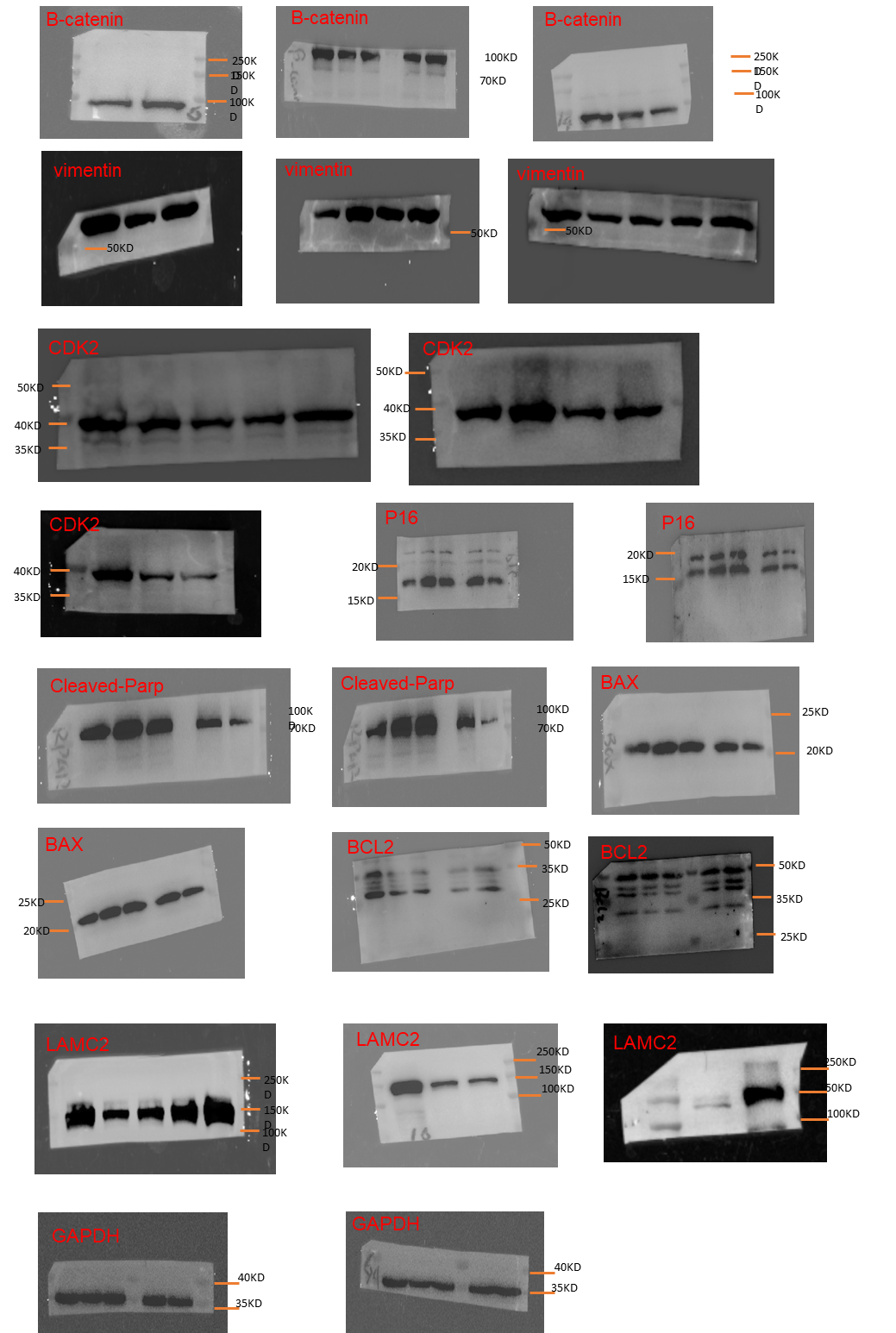


Figure 3D


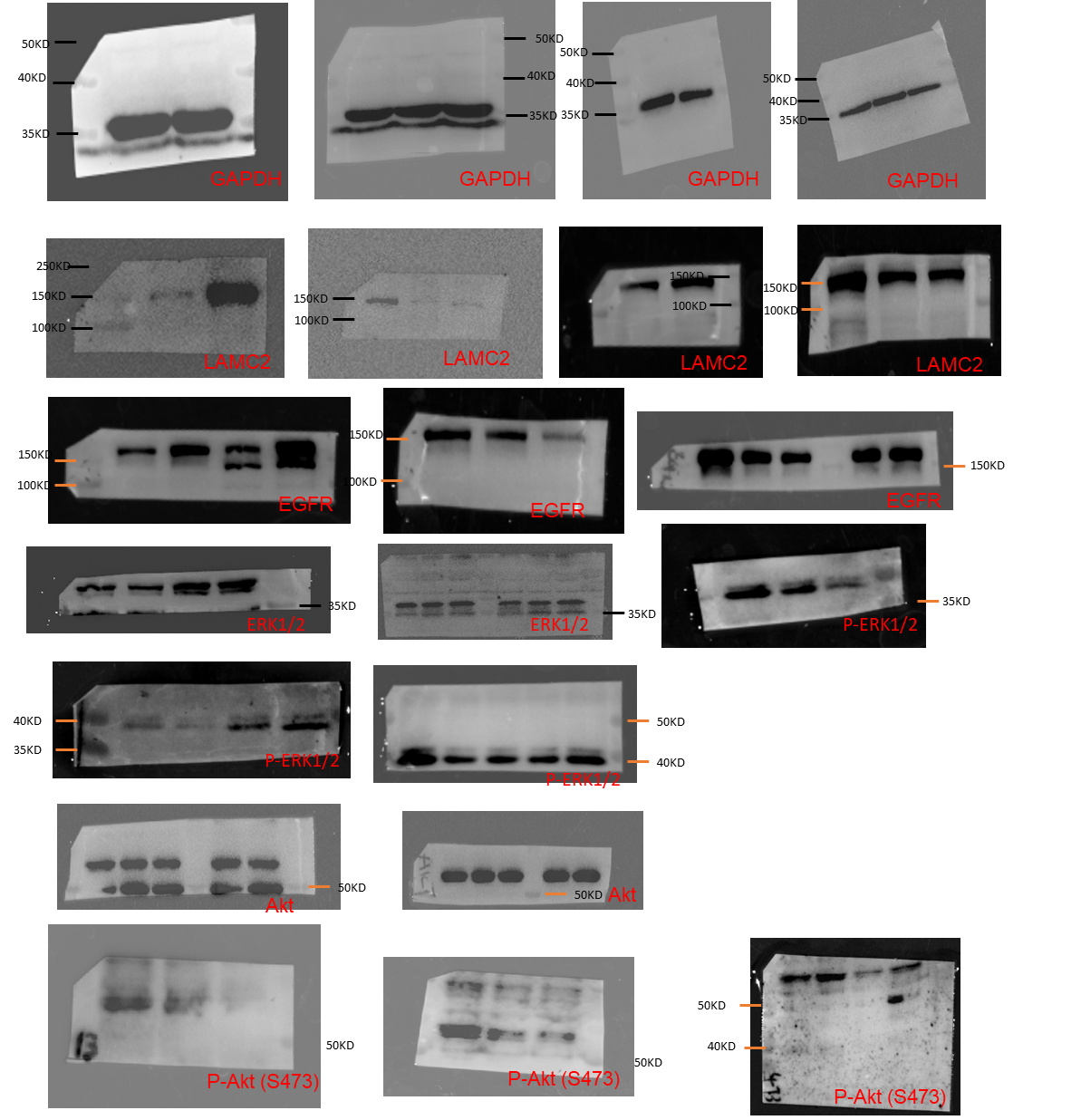


Figure 3K


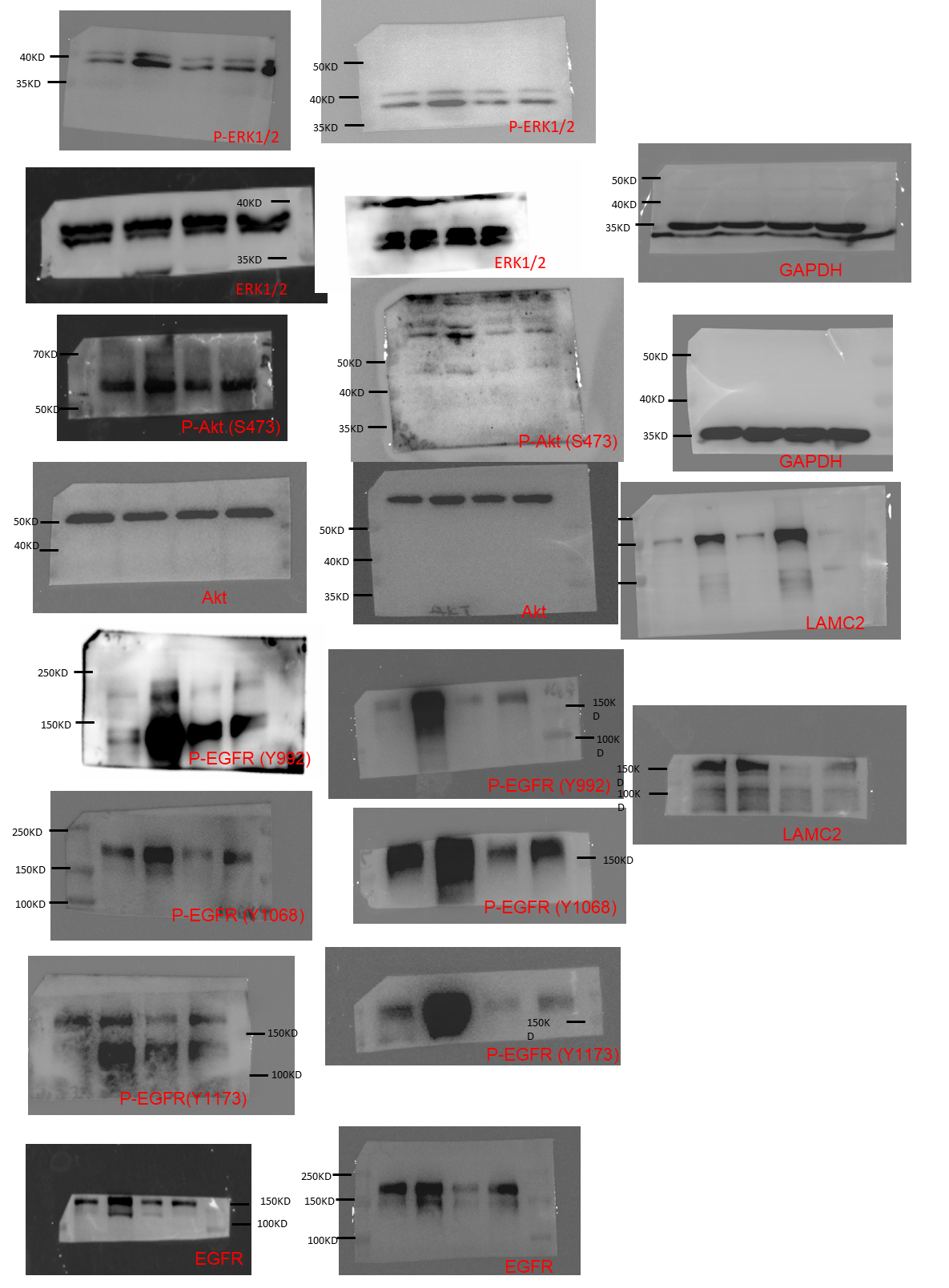


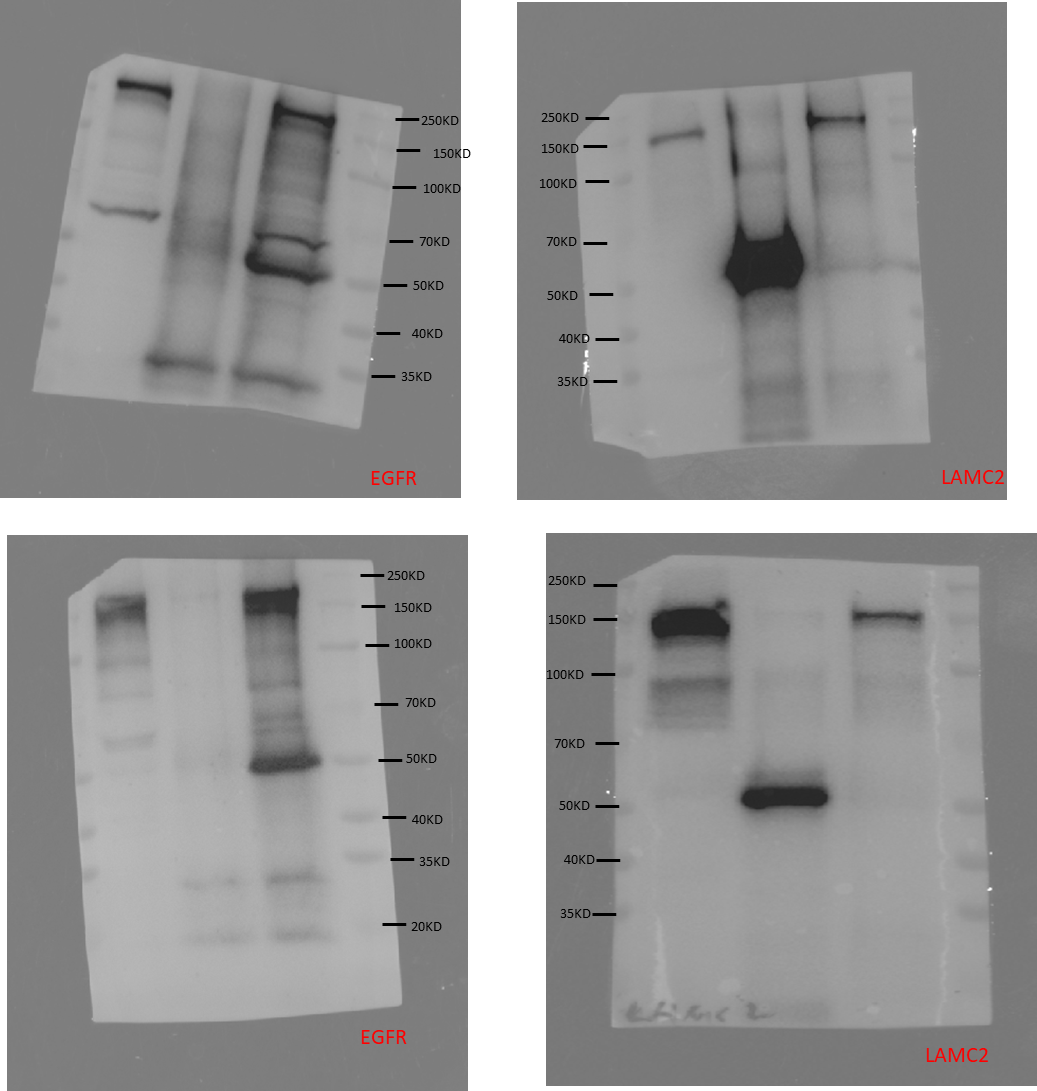
Figure 5A


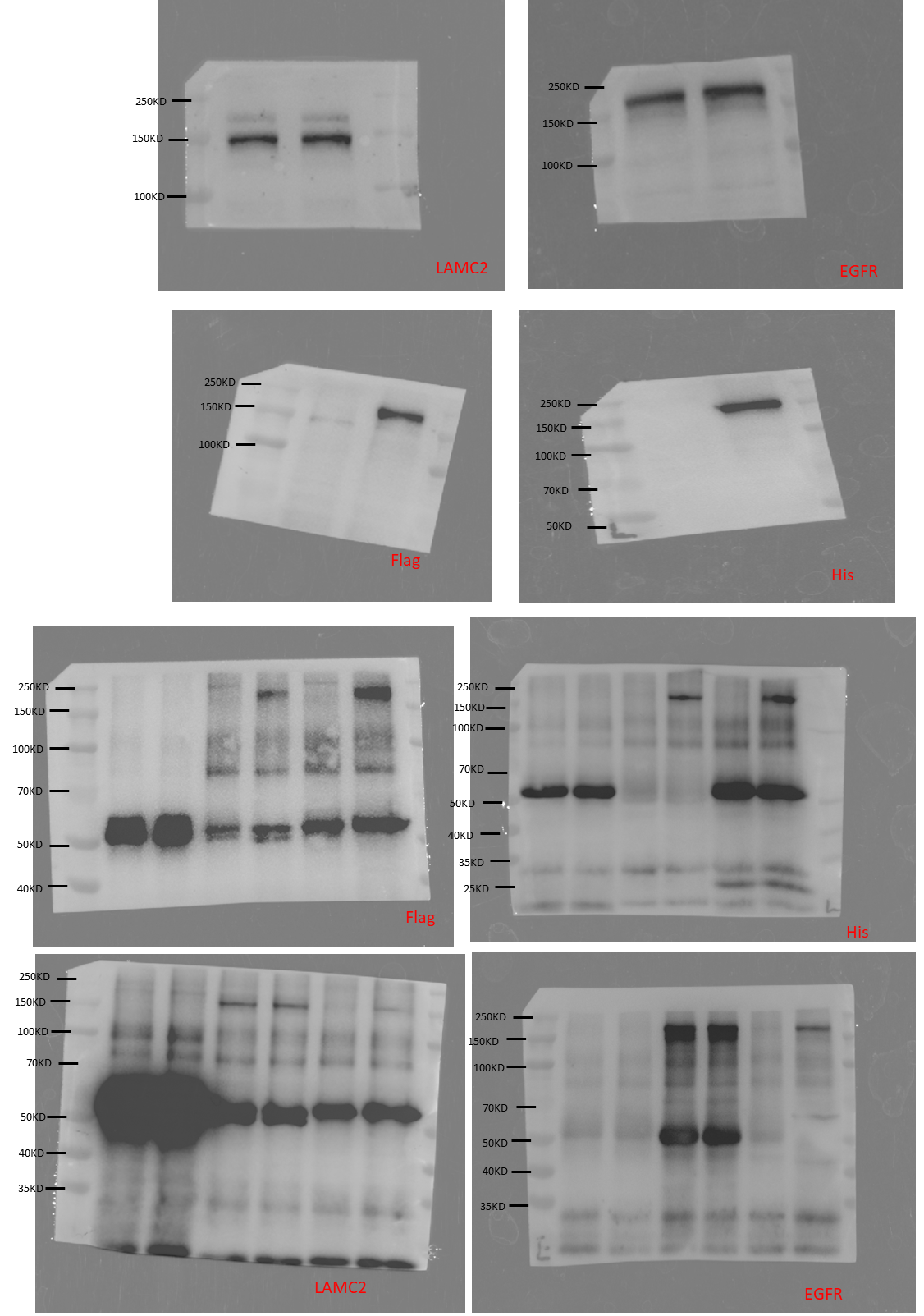
Figures 5C-D


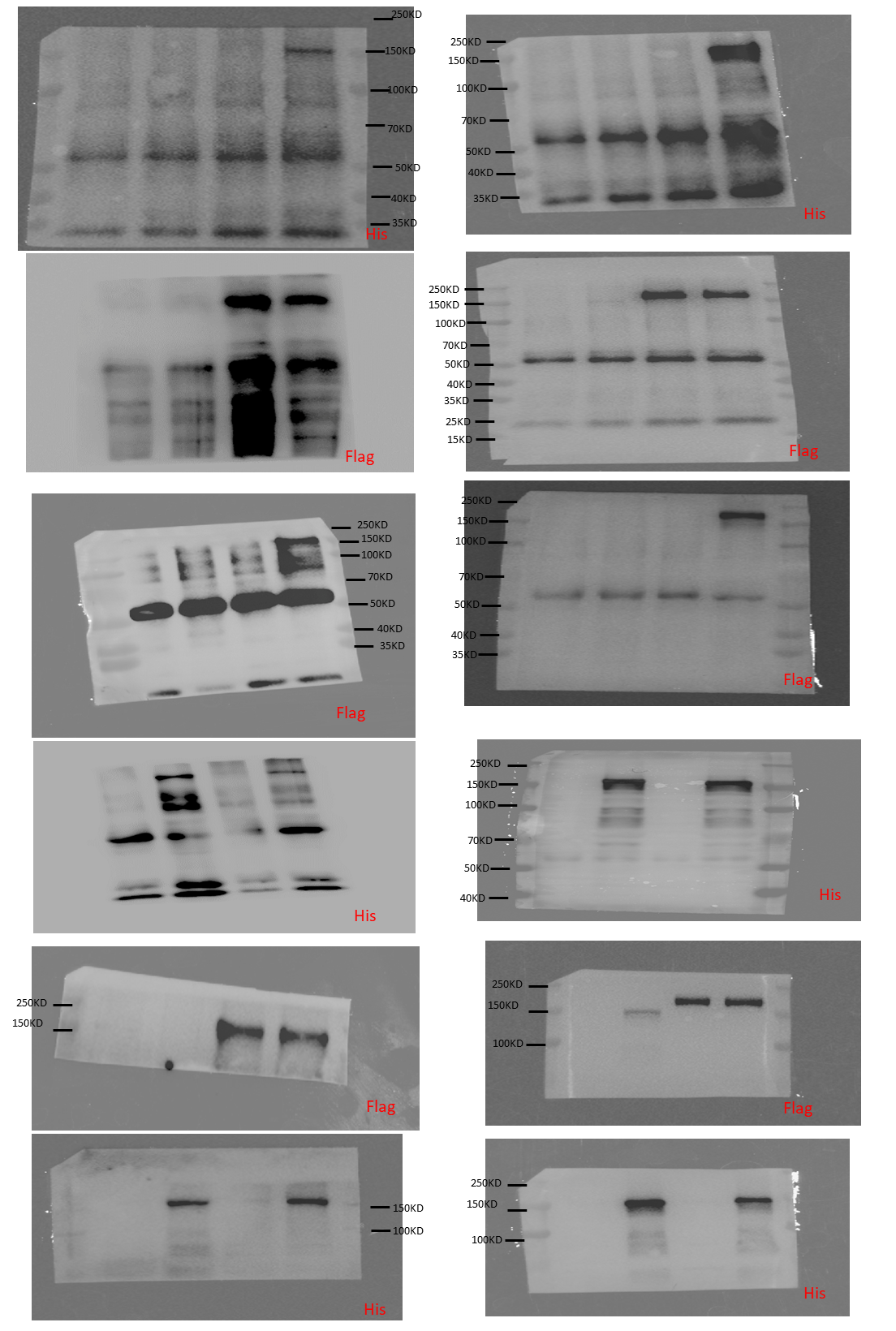
Figure 5E


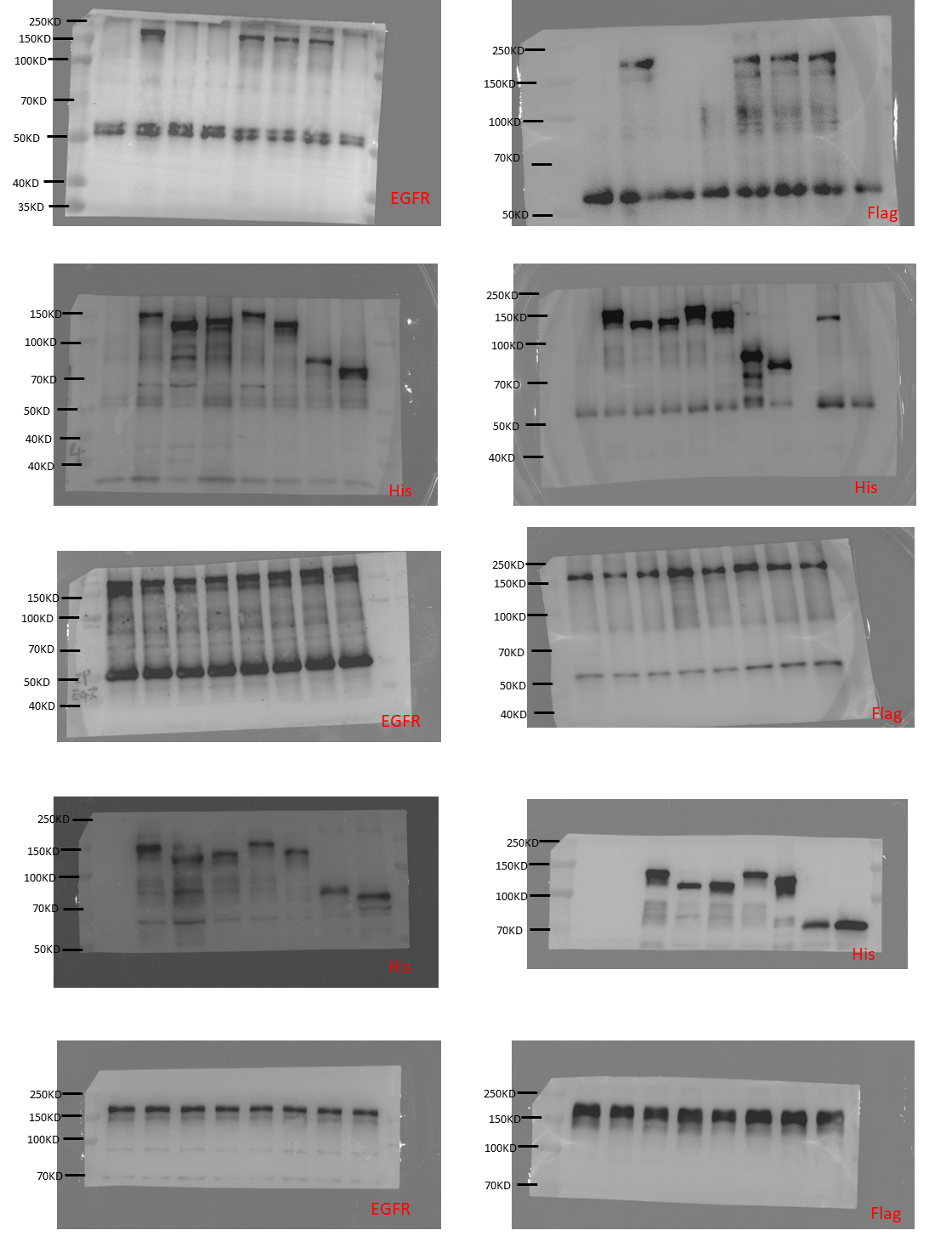
Figure 5I


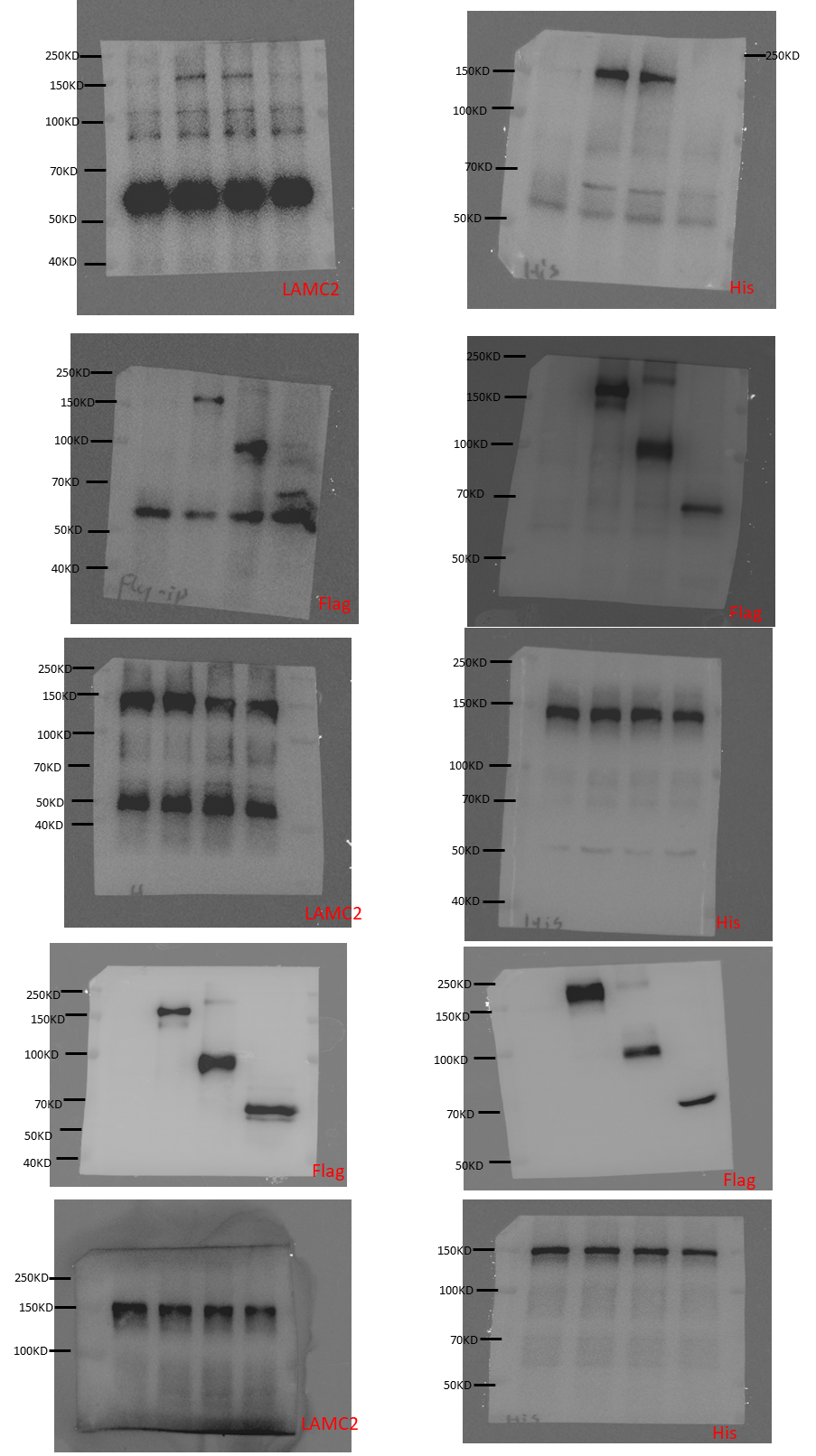
Figure 5K

Figure 6A


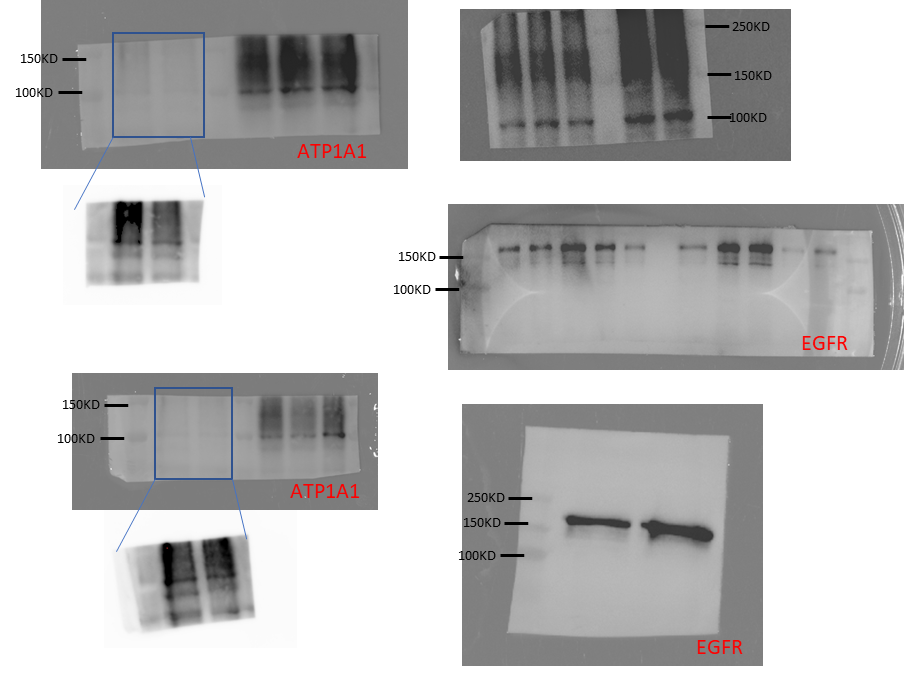


Figure 6C


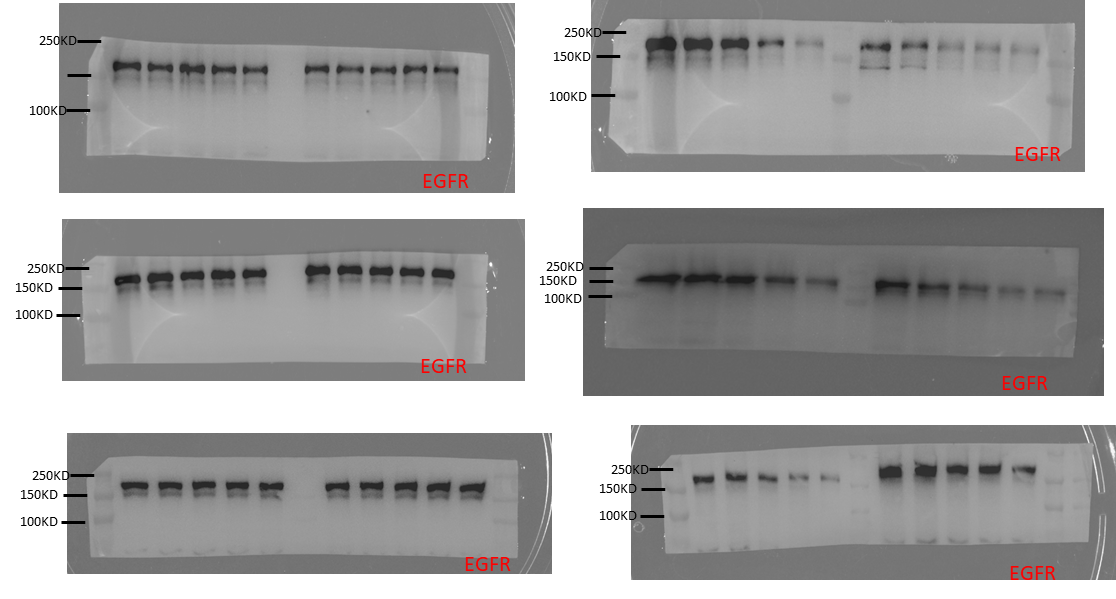


Figure 6D


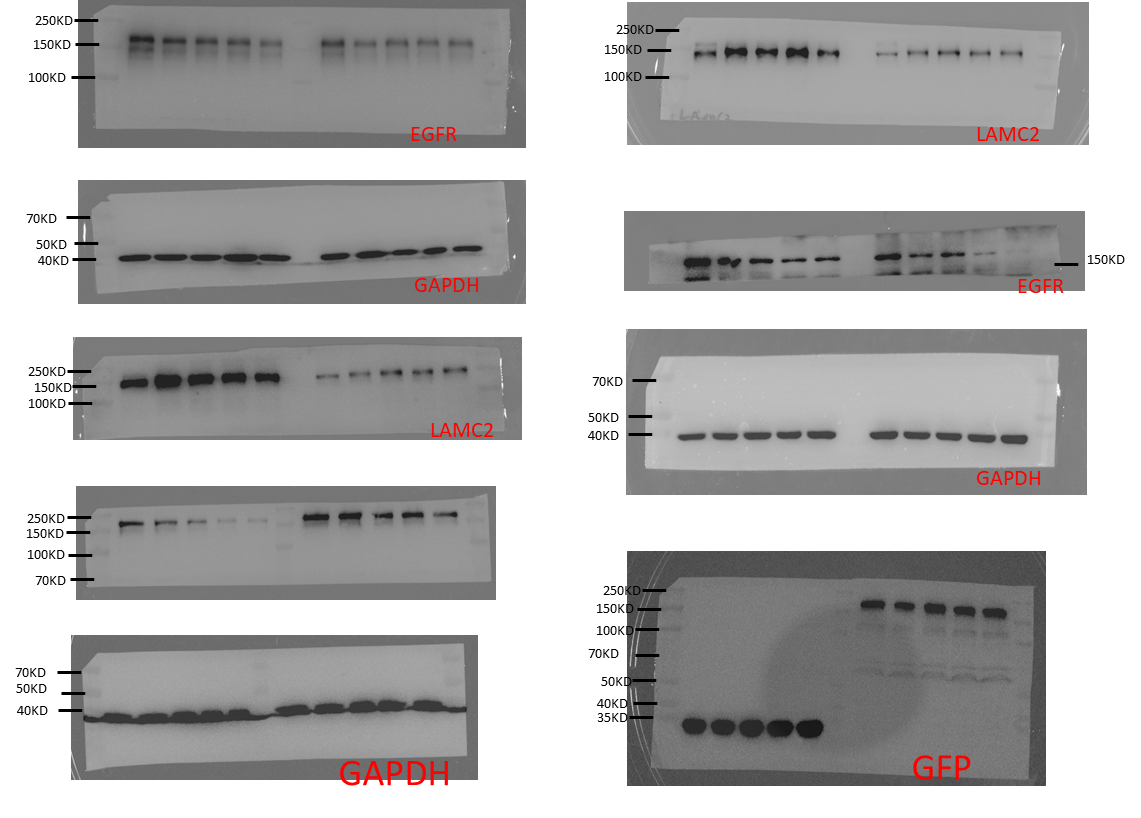


Figure 6G


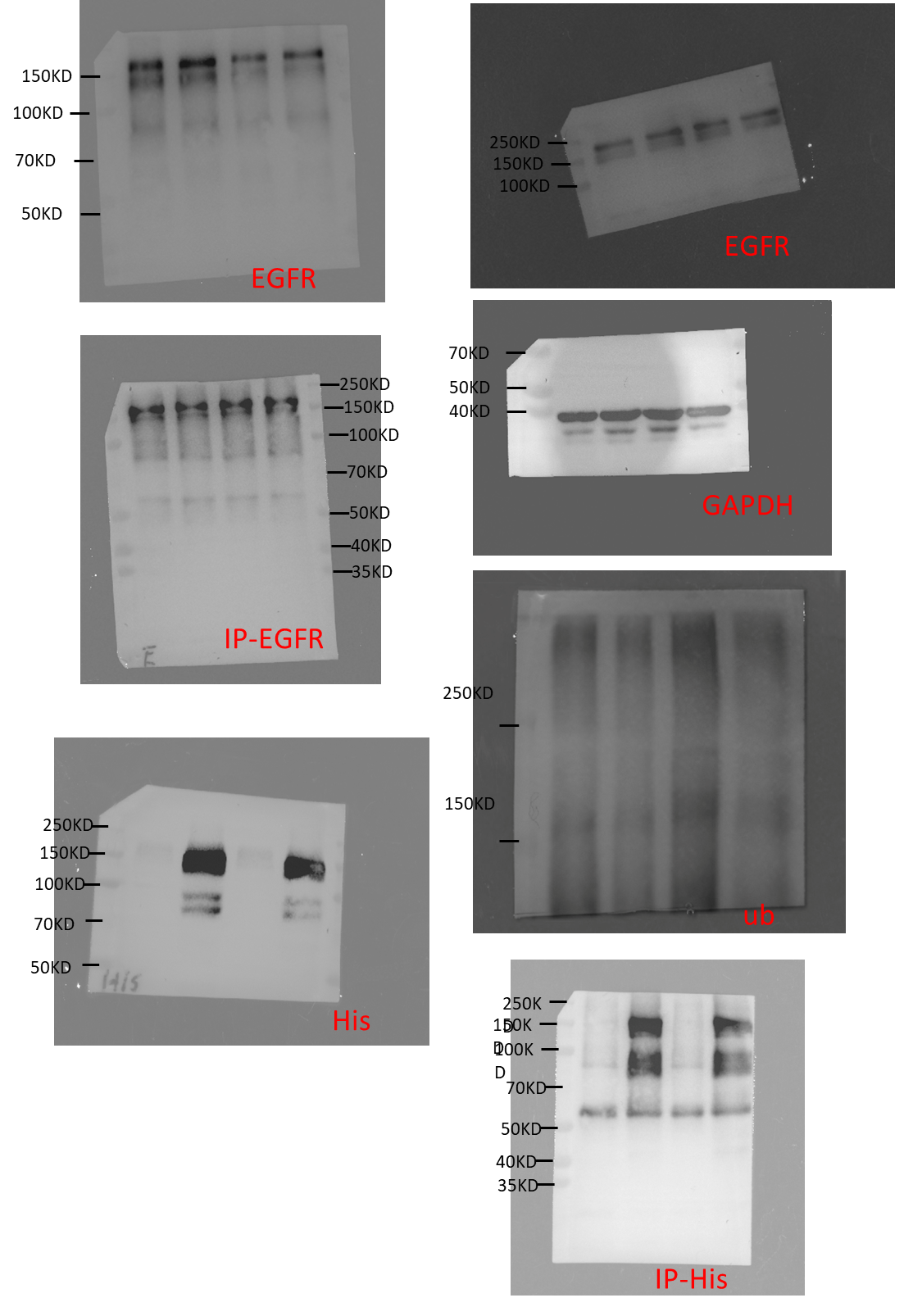


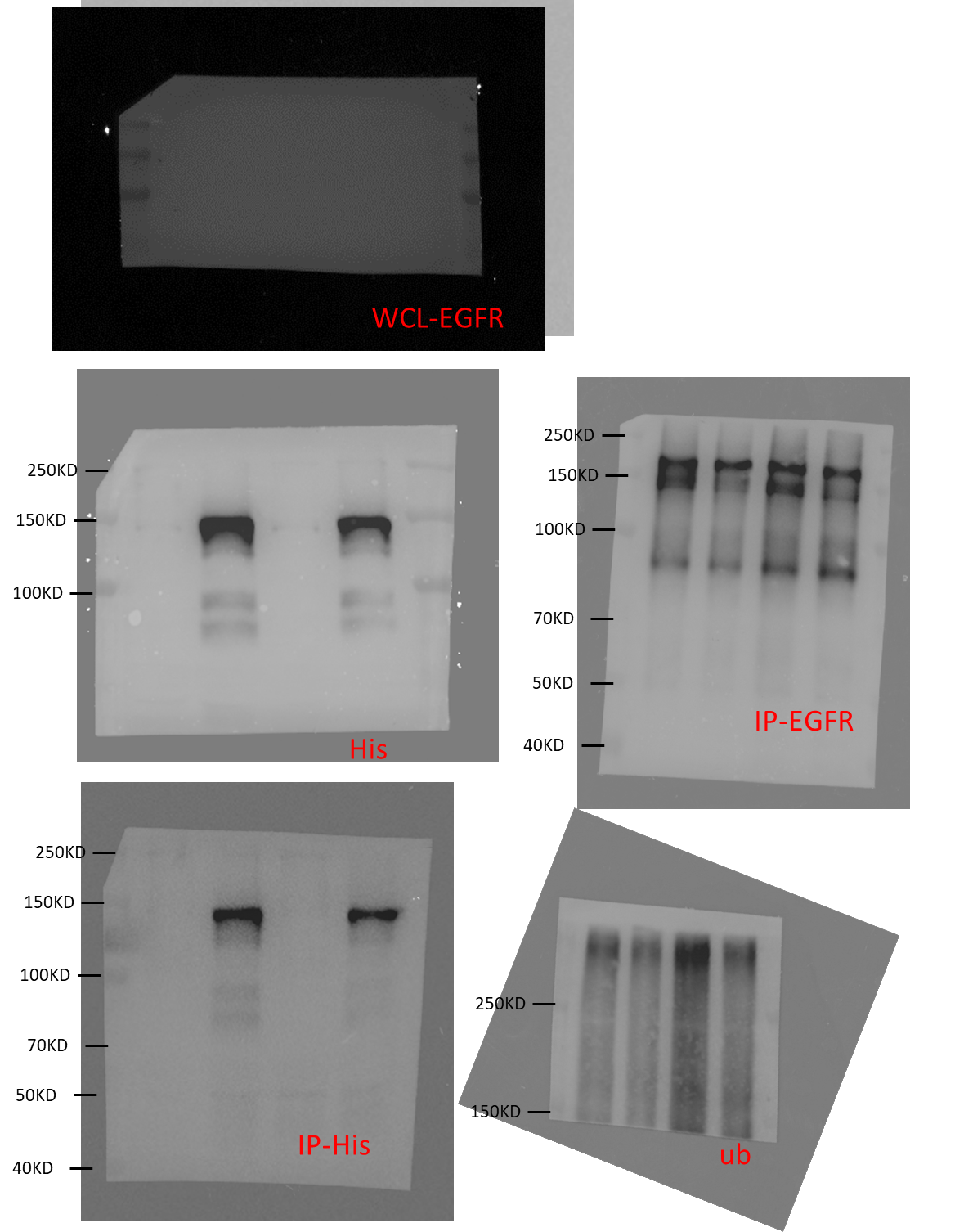
Figure 6H

Figure 6I


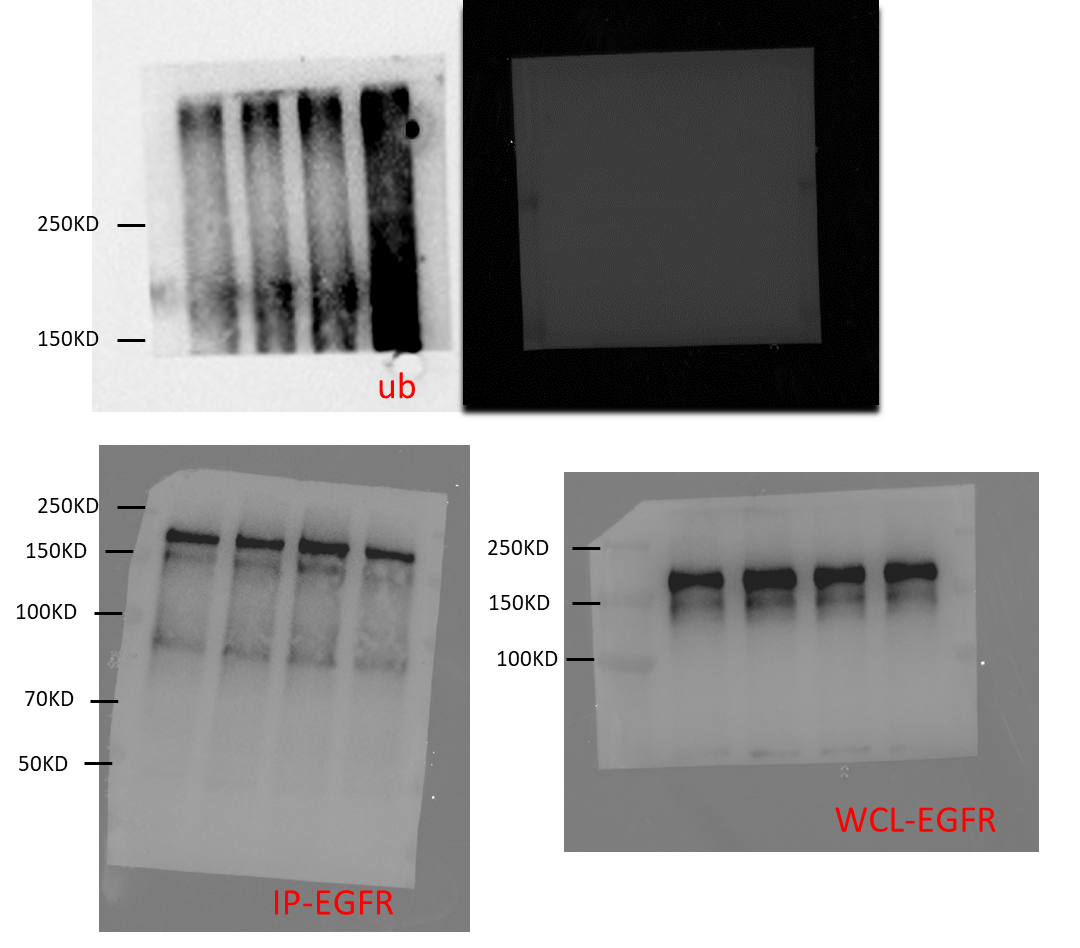


Figure 6J


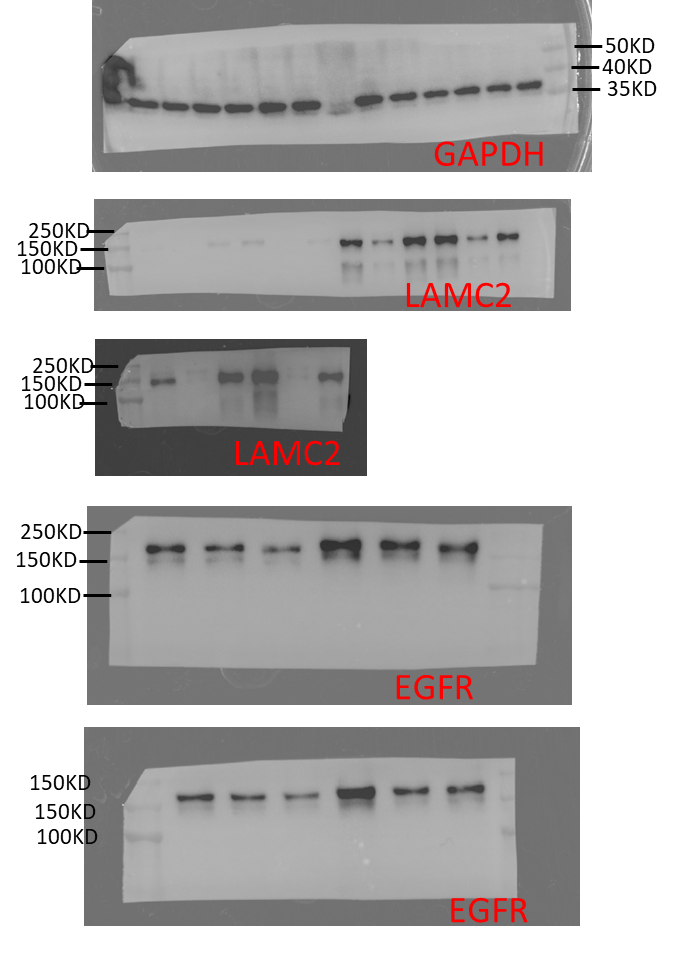


Figure 7


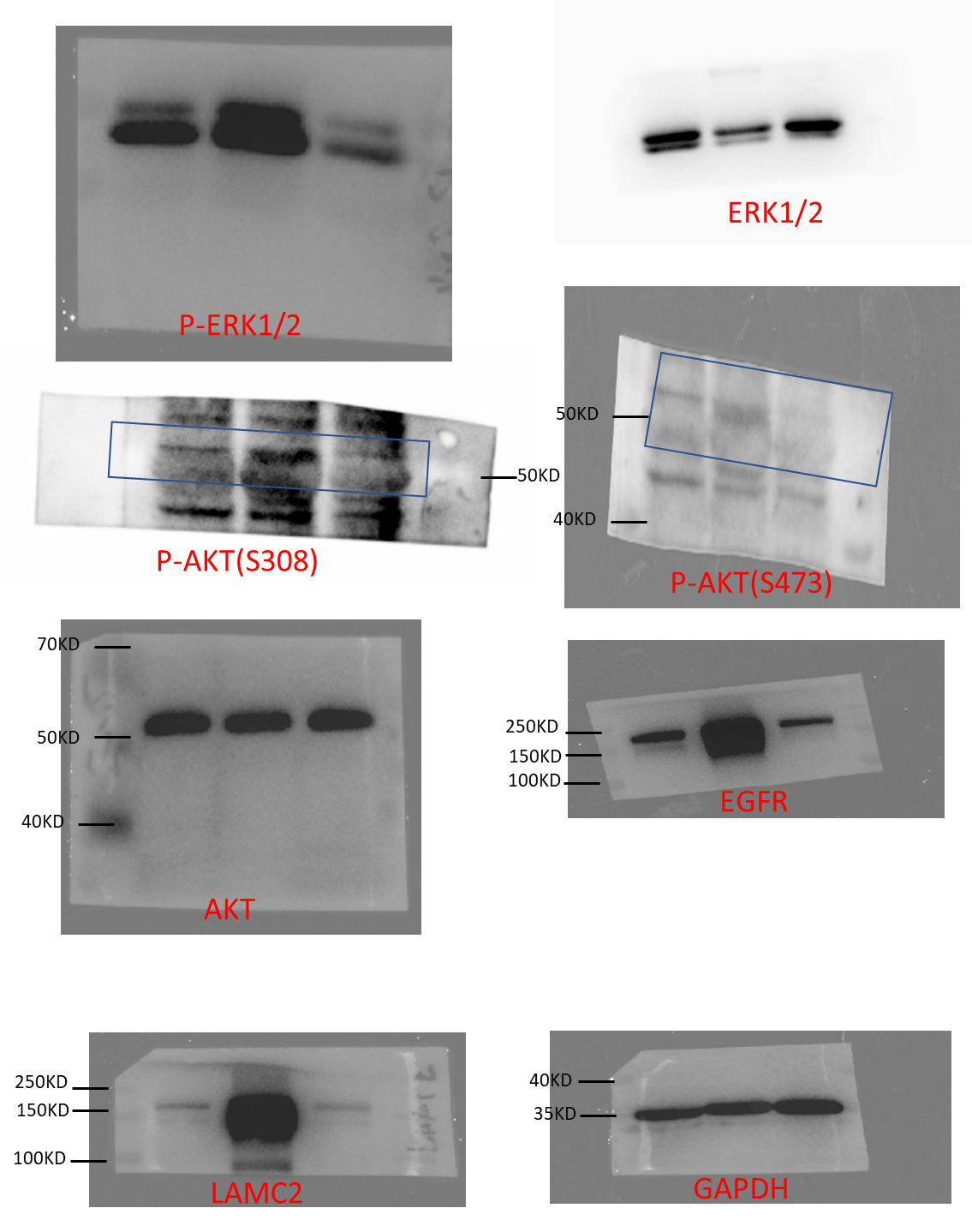

Supplement: Supplementary file 1 — wb raw data [file 41417_2023_654_MOESM1_ESM.docx]
