# Supplementary material for: LAMC2 promotes EGFR cell membrane localization and acts as a novel biomarker for tyrosine kinase inhibitors (TKIs) sensitivity in lung cancer
Source: Cancer Gene Ther. 2023 Aug 4;30(11):1498–512. doi: 10.1038/s41417-023-00654-7 (PMC10645587; doi:10.1038/s41417-023-00654-7)
Supplement: Supplementary file 2 — Supplementary Tables [file 41417_2023_654_MOESM2_ESM.docx]

**Supplementary Tables**

Table 1. Si-RNA information

| **Name** | **Sequence** |
| --- | --- |
| Negative siRNA (NC-siRNA) | 5'-TTCTCCGAACGTGTCACGT-3' |
| si-LAMC2-1 | 5'-GAAGCTTCCTTGGGAAACA-3' |
| si-LAMC2-2 | 5'-GTCAAAGCCTGTCCTTTGA-3' |
| Negative siRNA (NC-siRNA) | 5'-TTCTCCGAACGTGTCACGT-3' |
| si-EGFR-1 | 5'-GACAUAGUCAGCAGUGACU-3' |
| si-EGFR-2 | 5'-GAUCUUUCCUUCUUAAAGA-3' |

Table 2. Antibody information

| **Antibody** | **Company** | **Catalog No.** |
| --- | --- | --- |
| EGFR | Proteintech | 66455-1-Ig |
| EGFR | Proteintech | 18986-1-AP |
| LAMC2 | Abcam | ab210959 |
| His | Proteintech | 66005-1-Ig |
| Flag | Proteintech | 66008-4-Ig |
| GFP | abways | AB0005 |
| GRP78 | Proteintech | 11587-1-AP |
| GAPDH | abways | AB0038 |
| P-ERK1/2 | Cell signaling technology | #9101 |
| ERK1/2 | Proteintech | 11257-1-AP |
| P-Akt (S473) | Cell signaling technology | #4060 |
| AKT | abways | CY5561 |
| P-EGFR(Y992) | Genetex | GTX133429 |
| P-EGFR(Y1068) | Genetex | GTX25644 |
| P-EGFR(Y1173) | Genetex | GTX133437 |
| E-cadherin | Proteintech | 20874-1-AP |
| Vimentin | Proteintech | 10366-1-AP |
| CDK2 | Proteintech | 10122-1-AP |
| P16 | Proteintech | 10883-1-AP |
| Cleaved-parp | Cell signaling technology | #5625 |
| BAX | Proteintech | 0599-2-Ig |
| BCL2 | Proteintech | 12789-1-AP |
| Ki67 | Proteintech | 27309-1-AP |
