# Supplementary material for: LAMC2 promotes EGFR cell membrane localization and acts as a novel biomarker for tyrosine kinase inhibitors (TKIs) sensitivity in lung cancer
Source: Cancer Gene Ther. 2023 Aug 4;30(11):1498–512. doi: 10.1038/s41417-023-00654-7 (PMC10645587; doi:10.1038/s41417-023-00654-7)
Supplement: Supplementary file 3 — Supplementary Figures [file 41417_2023_654_MOESM3_ESM.pdf]

Supplementary Figure 1

A

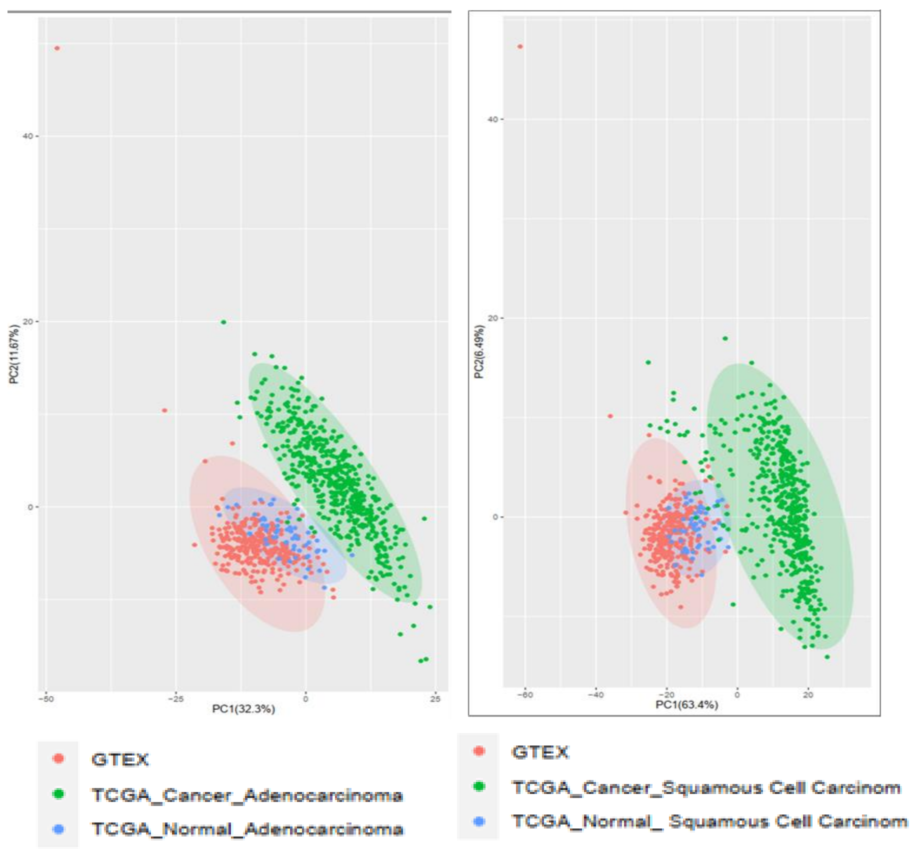

B

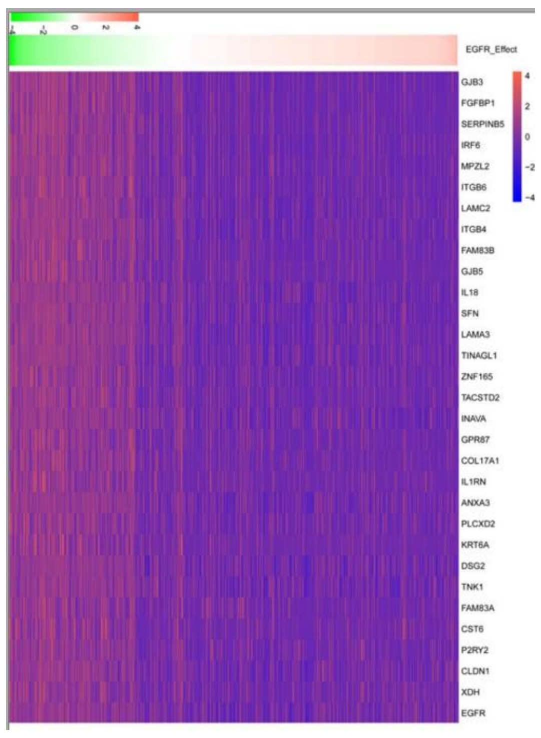

C

NAME	ES	NES	NOM p-val	FDR q-val
KEGG_BLADDER_CANCER	0.579798	1.926487	0	0.322071
KEGG_REGULATION_OF_ACTIN_CYTOSKELETON	0.530592	1.920934	0	0.165499
KEGG_ECM_RECEPTOR_INTERACTION	0.663345	1.920658	0	0.11071
KEGG_PANCREATIC_CANCER	0.571922	1.875285	0	0.125094
KEGG_FOCAL_ADHESION	0.574676	1.868009	0	0.104808
KEGG_SMALL_CELL_LUNG_CANCER	0.577011	1.860115	0	0.091898
KEGG_PATHWAYS_IN_CANCER	0.502135	1.837537	0	0.092602
KEGG_ADHERENS_JUNCTION	0.567894	1.812296	0	0.098529
KEGG_AXON_GUIDANCE	0.55153	1.807036	0	0.091424
KEGG_PATHOGENIC_ESCHERICHIA_COLI_INFECTION	0.459582	1.792686	0.012526	0.092307
KEGG_RENAL_CELL_CARCINOMA	0.554582	1.78499	0.004082	0.091276
KEGG_ERBB_SIGNALING_PATHWAY	0.532668	1.765014	0.002096	0.100444
KEGG_MTOR_SIGNALING_PATHWAY	0.531701	1.75808	0.001934	0.098301
KEGG_CHRONIC_MYELOID_LEUKEMIA	0.528581	1.744514	0.002008	0.101702

D

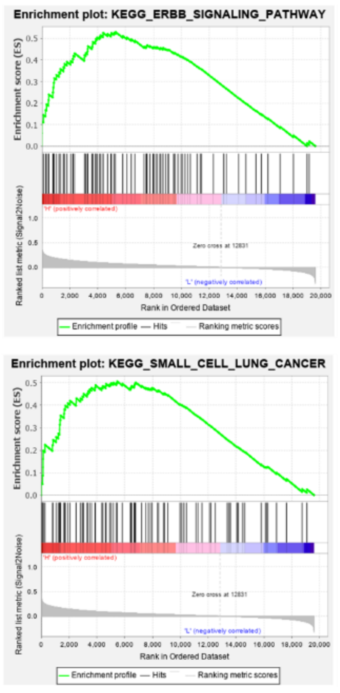

NAME	ES	NES	NOM p-va	FDR q-val
KEGG_CYTOSOLIC_DNA_SENSING_PATHWAY	0.567903	1.994005	0.001957	0.14368
KEGG_NATURAL_KILLER_CELL_MEDIATED_CYTOTOXICITY	0.614939	1.941876	0.001976	0.118898
KEGG_TOLL_LIKE_RECEPTOR_SIGNALING_PATHWAY	0.582825	1.89173	0	0.126335
KEGG_FOCAL_ADHESION	0.562751	1.87141	0.001976	0.115529
KEGG_NOD_LIKE_RECEPTOR_SIGNALING_PATHWAY	0.624739	1.869613	0.006012	0.09331
KEGG_RIG_I_LIKE_RECEPTOR_SIGNALING_PATHWAY	0.519557	1.869416	0.003922	0.078085
KEGG_APOPTOSIS	0.546312	1.845557	0.002058	0.079558
KEGG_JAK_STAT_SIGNALING_PATHWAY	0.538793	1.836	0	0.075754
KEGG_ANTIGEN_PROCESSING_AND_PRESENTATION	0.558174	1.827156	0.030888	0.073559
KEGG_REGULATION_OF_ACTIN_CYTOSKELETON	0.495299	1.82609	0	0.067549
KEGG_PANCREATIC_CANCER	0.554071	1.817539	0.002028	0.065823
KEGG_BLADDER_CANCER	0.54284	1.814992	0	0.061902
KEGG_CYTOKINE_CYTOKINE_RECEPTOR_INTERACTION	0.596471	1.813362	0.005952	0.058505
KEGG_PATHOGENIC_ESCHERICHIA_COLI_INFECTION	0.455401	1.812521	0.001919	0.054703
KEGG_ERBB_SIGNALING_PATHWAY	0.529721	1.795741	0.001972	0.058255
KEGG_ECM_RECEPTOR_INTERACTION	0.608184	1.777485	0.003766	0.0645
KEGG_LEUKOCYTE_TRANSENDOTHELIAL_MIGRATION	0.505444	1.705921	0.009766	0.107202
KEGG_VIRAL_MYOCARDITIS	0.586183	1.704014	0.023483	0.102756
KEGG_CHEMOKINE_SIGNALING_PATHWAY	0.523645	1.698236	0.016162	0.103171
KEGG_CHRONIC_MYELOID_LEUKEMIA	0.509589	1.696655	0.008048	0.099519
KEGG_ADHERENS_JUNCTION	0.516947	1.695471	0.010183	0.095842
KEGG_AXON_GUIDANCE	0.509697	1.688994	0.004107	0.097717
KEGG_P53_SIGNALING_PATHWAY	0.515128	1.685451	0.004024	0.096467
KEGG_LYSOSOME	0.462504	1.678458	0.025194	0.098806
KEGG_MAPK_SIGNALING_PATHWAY	0.452514	1.670325	0.002066	0.100186
KEGG_GLIOMA	0.498981	1.66998	0.007984	0.096575
KEGG_ENDOCYTOSIS	0.4476	1.667304	0.009823	0.095162
KEGG_MELANOMA	0.482682	1.661655	0.012	0.096315
KEGG_SMALL_CELL_LUNG_CANCER	0.506762	1.660656	0.006061	0.093972

Supplementary Figure 2

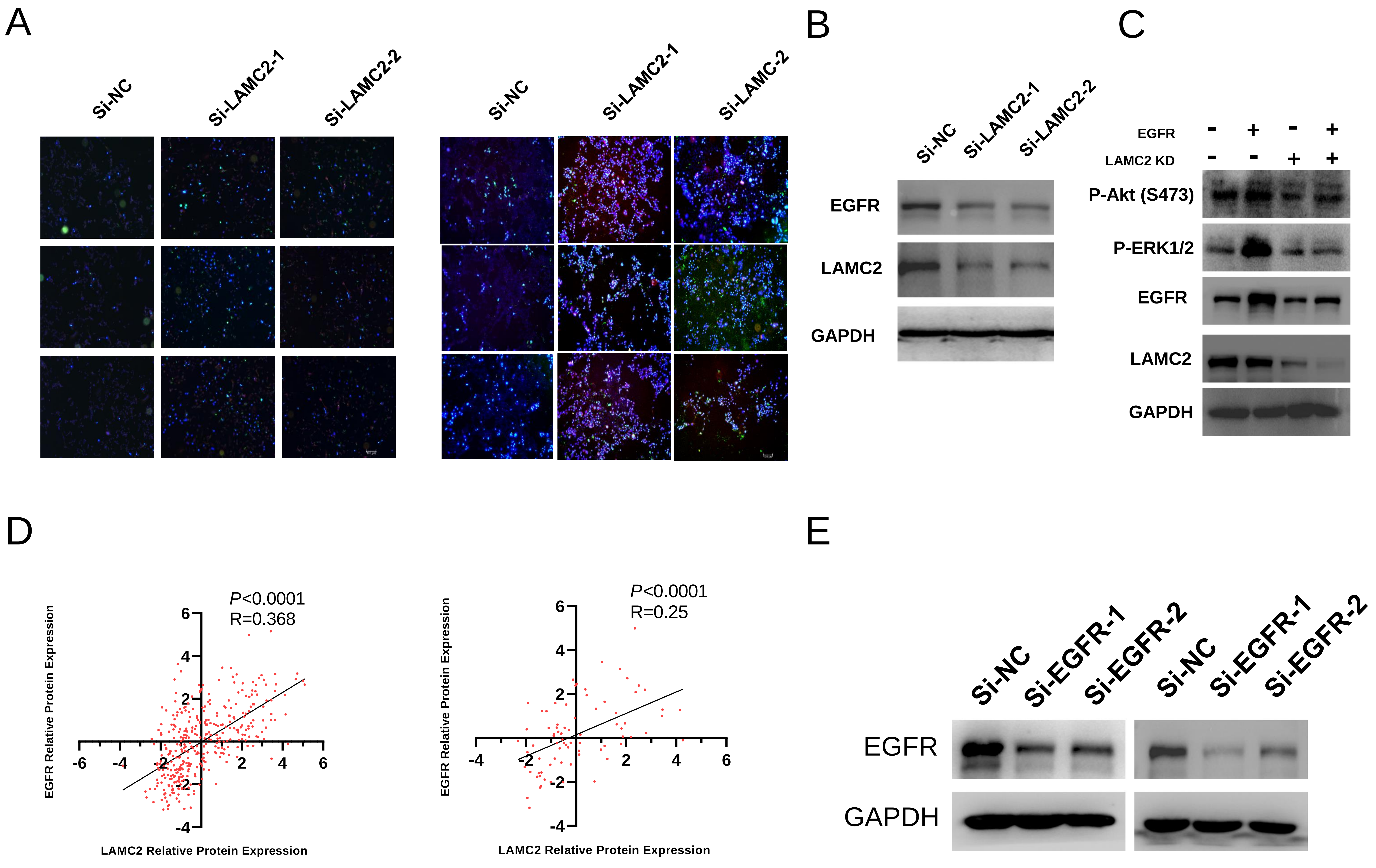

Supplementary Figure 3

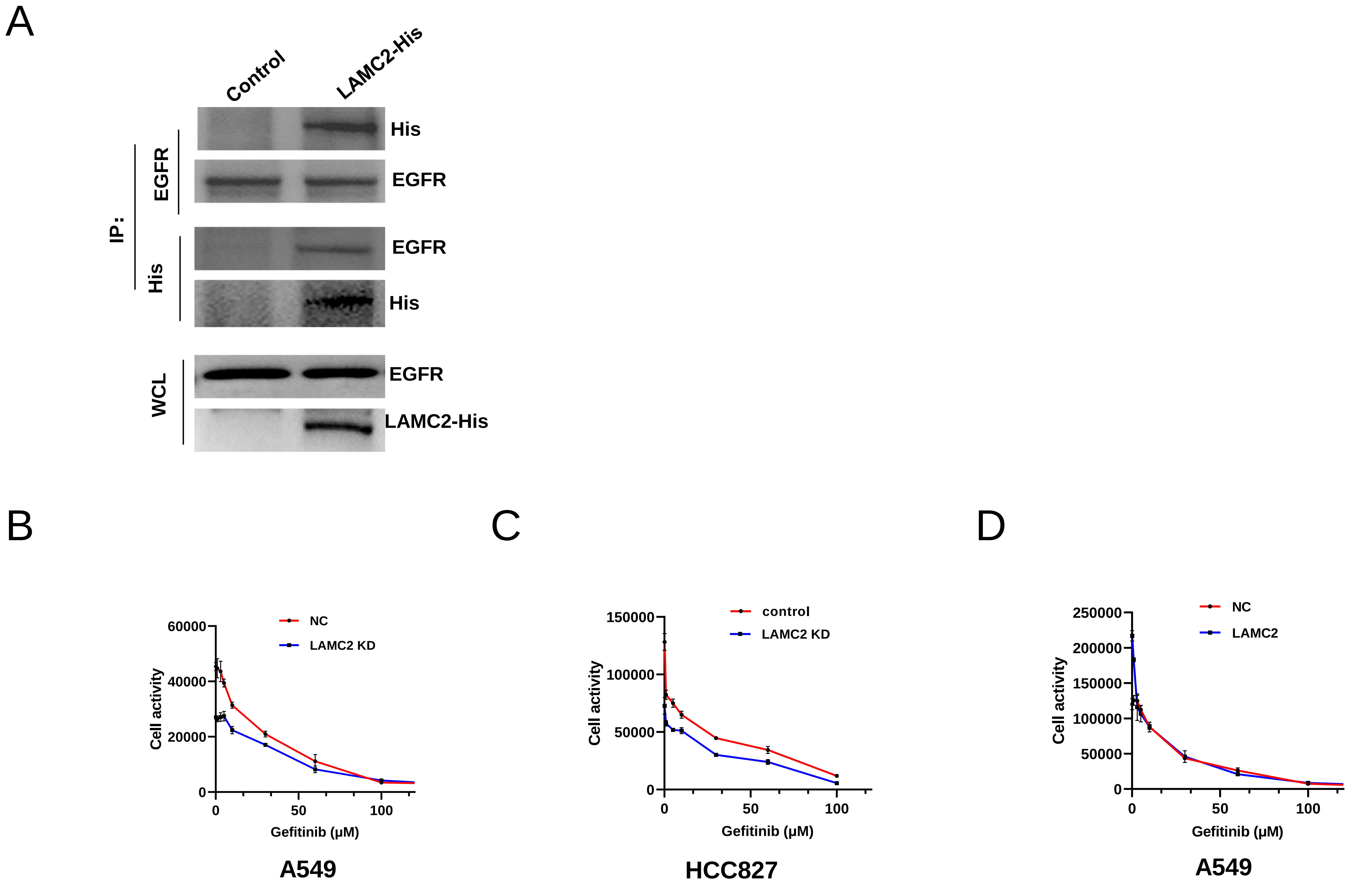

Supplementary Figure Legends

Supplementary Figure 1

(A) PCA analyses showed distinct clusters top 53 common sensitive genes in adenocarcinoma, squamous carcinoma, their respective controls, and GTEx control groups.

(B) Heatmap of top differentially expressed EGFR-dependent genes from CCLE and Project Achilles.

(C) KEGG pathway enrichment analysis showed significantly enriched pathways associated with LAMC2.

(D) GSEA analysis indicated FGFBP1 is significantly correlated with ERBB signaling pathway and small cell lung cancer (Left). KEGG pathway enrichment analysis showed significantly enriched pathways associated with FGFBP1 (Right).

Supplementary Figure 2

(A) Representative images of Hoechst, Annexin V, PI triple fluorescence staining (merge) showing A549 (left) and HCC827 (right) cell apoptosis after EGFR siRNA transfection.

(B) Western blotting analyses indicated protein levels of EGFR and LAMC2 in NCI-H23 cells transfected with LAMC2 siRNA.

(C) Western blotting analyses indicated protein levels of P-AKT(S473), P-ERK1/2, EGFR, and LAMC2 in NCI-H23 cells co-transfected with EGFR plasmid and LAMC2 siRNA.

(D) Correlation graph generated from CCLE database demonstrated positive correlation between EGFR and LAMC2 protein expression levels in all cancer (left) and lung cancer (right).

(E) Western blotting analyses indicated protein levels of EGFR in A549 (left) and HCC

827(right) cells after transfection with EGFR siRNA.

Supplementary Figure 3

(A) Co-IP analyses were used to detect protein interaction between exogenous LAMC2 and EGFR in NCI-H23 cells. Western blotting analysis using WCL to detect EGFR and LAMC2-His, bottom.

(B-D) Cell activity levels of A549 (B) and NCI-H23 (C) cells transfected with LAMC2 siRNA and HCC827 (D) cells transfected with LAMC2 overexpression vector after Gefitinib treatment.
